# Direct Imaging of Plant Metabolites in the Rhizosphere Using Laser Desorption Ionization Ultra-High Resolution Mass Spectrometry

**DOI:** 10.3389/fpls.2021.753812

**Published:** 2021-12-03

**Authors:** Martin Lohse, Rebecca Haag, Eva Lippold, Doris Vetterlein, Thorsten Reemtsma, Oliver J. Lechtenfeld

**Affiliations:** ^1^Department of Analytical Chemistry, Helmholtz Centre for Environmental Research – UFZ, Leipzig, Germany; ^2^Ansbach University of Applied Sciences, Ansbach, Germany; ^3^Department of Soil System Science, Helmholtz Centre for Environmental Research – UFZ, Halle, Germany; ^4^Soil Science, Martin Luther University Halle-Wittenberg, Halle, Germany; ^5^Institute of Analytical Chemistry, University of Leipzig, Leipzig, Germany; ^6^ProVIS – Centre for Chemical Microscopy, Helmholtz Centre for Environmental Research – UFZ, Leipzig, Germany

**Keywords:** chemical imaging, spatial metabolomics, low molecular weight organics, root exudation, biogeochemical gradients, FT-ICR-MS, *Zea mays* L

## Abstract

The interplay of rhizosphere components such as root exudates, microbes, and minerals results in small-scale gradients of organic molecules in the soil around roots. The current methods for the direct chemical imaging of plant metabolites in the rhizosphere often lack molecular information or require labeling with fluorescent tags or isotopes. Here, we present a novel workflow using laser desorption ionization (LDI) combined with mass spectrometric imaging (MSI) to directly analyze plant metabolites in a complex soil matrix. Undisturbed samples of the roots and the surrounding soil of *Zea mays* L. plants from either field- or laboratory-scale experiments were embedded and cryosectioned to 100 μm thin sections. The target metabolites were detected with a spatial resolution of 25 μm in the root and the surrounding soil based on accurate masses using ultra-high mass resolution laser desorption ionization Fourier-transform ion cyclotron resonance mass spectrometry (LDI-FT-ICR-MS). Using this workflow, we could determine the rhizosphere gradients of a dihexose (e.g., sucrose) and other plant metabolites (e.g., coumaric acid, vanillic acid). The molecular gradients for the dihexose showed a high abundance of this metabolite in the root and a strong depletion of the signal intensity within 150 μm from the root surface. Analyzing several sections from the same undisturbed soil sample allowed us to follow molecular gradients along the root axis. Benefiting from the ultra-high mass resolution, isotopologues of the dihexose could be readily resolved to enable the detection of stable isotope labels on the compound level. Overall, the direct molecular imaging *via* LDI-FT-ICR-MS allows for the first time a non-targeted or targeted analysis of plant metabolites in undisturbed soil samples, paving the way to study the turnover of root-derived organic carbon in the rhizosphere with high chemical and spatial resolution.

## Introduction

The rhizosphere is a hotspot for microbial activity, organic carbon input, and carbon turnover in soils. The interlinked physical, chemical, and biological processes in the rhizosphere combined with the structural heterogeneity of soils are a challenge for the analysis of this complex system. The interaction of root exudates with microbes (Haichar et al., 2008; Zhalnina et al., 2018), the effect of exudation on soil structure and aggregation (Baumert et al., 2018), and the association of molecules to minerals (Keiluweit et al., 2015; Li et al., 2021) result in a highly variable and permanently changing distribution of molecules. While the direct sampling of root exudates requires artificial – mostly soil-free – conditions (Phillips et al., 2008; Oburger and Jones, 2018) the determination of spatial gradients of small and often polar and mobile molecules in the soil cannot be easily achieved by *ex situ* sampling of exudates. The temporal dynamics of root exudation in the soil can be studied by sampling the soil solution (Dessureault-Rompré et al., 2007; Schulz and Vetterlein, 2007; Weidenhamer et al., 2014; Tiziani et al., 2021) and combined with structural information about the root-soil system *via* X-ray computed tomography (CT) (Lohse et al., 2020). However, small-scale spatial heterogeneity of root exudates within a few hundred micrometers cannot be analyzed by these approaches.

Detailed information on the spatial distribution of root-derived metabolites in soils can be obtained by molecular imaging methods. A variety of analytical techniques allows the *in situ* detection of rhizosphere molecular gradients without separating the bulk soil (BS) and rhizosphere soil (RS). While all methods used for the analysis of chemical gradients in the rhizosphere operate at specific spatial resolutions and can reveal different degrees of specific chemical information (Vetterlein et al., 2020), molecular mass spectrometric imaging (MSI) methods are especially suited to detect intact organic molecules with high chemical resolution. Matrix-assisted laser desorption ionization mass spectrometry (MALDI-MS) is routinely used for label-free biochemical imaging (Liebeke et al., 2015; Sturtevant et al., 2016). Since intact molecules in complex matrices can be determined with high sensitivity, as well as suitable spatial and chemical resolution, even complex processes such as metabolism can be visualized (Li et al., 2008; Dekker et al., 2015).

Despite these advantages, (MA)LDI-MS for the imaging of biogeochemical processes is rarely reported although previously applied to analyze lipid biomarkers in sediments (Wörmer et al., 2014, 2019; Alfken et al., 2019, 2020).

Molecular MSI *via* (MA)LDI-MS has been demonstrated for isolated roots (Jun et al., 2010; Peukert et al., 2012; Hölscher et al., 2014; Rudolph-Mohr et al., 2015; Sarabia et al., 2018; Korenblum et al., 2020; Veličković et al., 2020a; Döll et al., 2021), root nodules (Ye et al., 2013), the ginger rhizome (Harada et al., 2009), and bacterial micro-colonies on the root surface (Debois et al., 2014; Pessotti et al., 2019). Derivatization reactions can be used to increase the sensitivity for the detection of metabolites in maize roots during the MSI (Dueñas et al., 2019; O’Neill and Lee, 2020). Recently, indirect rhizosphere molecular MSI utilizing a membrane to extract the molecules from the root-soil interface has been demonstrated (Veličković et al., 2020b).

To date, no direct (MA)LDI-MS analysis of the rhizosphere has been reported. The high spatial resolution offered by MSI would be a useful tool to better understand the feedback loops between the soil, plants, and microbes, to characterize root-derived carbon input and organic matter transformation in the soil and its effect on nutrient cycling and soil structure formation (Vetterlein et al., 2020). In combination with molecular information, transformations and biogeochemical interactions in the rhizosphere can be visualized and may give an insight into metabolic processes (Dijkstra et al., 2011).

Compared with secondary ion mass spectrometry (SIMS), MALDI and laser desorption ionization (LDI) are considered soft ionization techniques and allow for a high spatial resolution ranging from 10 μm (Harada et al., 2009; Hölscher et al., 2014; Pessotti et al., 2019) to 50 μm (Rudolph-Mohr et al., 2015; Korenblum et al., 2020). This spatial resolution would allow the detection of metabolite gradients in the rhizosphere covering a few hundred micrometers (Holz et al., 2019; Bilyera et al., 2021) up to several mm (Schenck zu Schweinsberg-Mickan et al., 2010; Holz et al., 2018b). Ultra-high mass resolution mass spectrometers such as the Fourier-transform ion cyclotron resonance mass spectrometry (FT-ICR-MS) and the Orbitrap mass spectrometry have sufficient mass resolving power to unambiguously assign nitrogen- and sulfur-containing molecules, as well as to resolve isotopologues within complex mixtures (Hawkes et al., 2016). Consequently, the combination of (MA)LDI with FT-ICR-MS is a powerful tool to detect biogeochemical gradients (Wörmer et al., 2014; Veličković et al., 2020b).

Here we present a workflow for the direct analysis of the molecular gradients in the rhizosphere. To directly analyze the molecular distribution in the proximity of a root, several conditions have to be met:

• The sampling and sample preparation strategy has to preserve the structural integrity of the root-soil system, even in the vacuum of the ion source (Mueller et al., 2013; Veličković et al., 2020b; Bandara et al., 2021).
• The chemical composition of the potential target molecules must not be altered by agents used for fixation, embedding media, or solvents that could selectively remove compounds (Alfken et al., 2019).
• The method shall provide sufficient spatial resolution to detect root-induced carbon input and a high chemical resolution to unambiguously resolve masses and assign formulas within the omnipresent complex soil organic matter (SOM) matrix.

The presented novel sample preparation and measurement workflow was applied to *Zea mays* L. roots and the surrounding soil from the field- and laboratory-scale experiments. After gelatin embedding, cryosectioning, and measurement *via* LDI-FT-ICR-MS, the spatial distribution of the plant metabolites directly in the soil was recovered.

## Materials and Methods

### Chemicals

Gelatin from bovine (Sigma-Aldrich, St. Louis, MO, United States), sodium carboxymethyl cellulose (CMC) (low viscosity, Sigma-Aldrich), and a Tissue-Tek^®^ optimal cutting temperature (OCT) compound (Sakura Finetek, Alphen aan den Rijn, Netherlands) were used for the sample preparation. Sucrose (Pharmaceutical Secondary Standard, Certified Reference Material, Sigma-Aldrich) was purchased to generate the reference spectra. The organic matter reference sample Suwannee River Fulvic Acid (SRFA II, International Humic Substances Society) was used for quality control during FT-ICR-MS measurement. Ultrapure water (MQW) was generated from a MilliQ Integral five system (Merck, Darmstadt, Germany).

### Samples and Sampling

*Zea mays* L. plants (wild type, B73 cultivar) were grown in field- and laboratory-scale experiments. Metal cylinders (*h* = 2 cm, *d* = 0.74–0.91 cm, wall thickness = 400 μm, sharpened) were used to punch out an undisturbed soil sample (1.0 cm height) with roots (undisturbed refers to the soil structure and the spatial relationship between the root surface and soil particle arrangement). Experimental details on the field (Vetterlein et al., 2021) and laboratory experiments can be found in the Supplementary Material to this article (Supplementary Table 1).

The field samples were taken at BBCH 19 (Biologische Bundesanstalt, Bundessortenamt and Chemical industry) following the German coding of the phenological growth stages of maize (Lancashire et al., 1991; Bleiholder et al., 2001; Meier, 2018) from plots established in the Helmholtz Centre for Environmental Research (UFZ) Research Station Bad Lauchstädt, Germany (N°51.390424; E°11.875933), where a hole was excavated and undisturbed soil samples were taken at a depth of 12 and 21 cm. The soil was a haplic Phaeozem (loam) from the vicinity of Schladebach in Saxony Anhalt, Germany (N°51.308725; E°12.104531) (Vetterlein et al., 2021).

To trace the fate of assimilated carbon in the rhizosphere, ^13^C pulse labeling was conducted on the day before sampling (Vetterlein et al., 2021). Gas-tight chambers with a volume of 0.42 m^3^ were set up and ^13^CO_2_ was produced by mixing Na_2_^13^CO_3_ (99% ^13^C Na_2_CO_3_ Euriso-Top GmbH, Germany) with a solution of 5 mol L^–1^ H_2_SO_4_ (95–97%, for analysis, EMSURE^®^ ISO, Merck KGaA, Darmstadt, Germany). Nine plants per chamber and field plot were labeled with two equal portions of 8.14 g of Na_2_^13^CO_3_ tracer over 4 h with the first pulse given after sunrise and the second pulse 2 h after. After the last application, the chamber was left for another 2 h to allow assimilation of ^13^CO_2_. The soil surface was sealed during ^13^C labeling with a plastic foil to prevent direct gas exchange.

The ^13^CO_2_ concentration in the gas-tight chamber was determined *via* gas chromatography (GC-Box, Thermo Fisher Scientific, Bremen, Germany, column: PoraPLOT Q, Agilent J&W) coupled with an isotope ratio mass spectrometer (Delta plus XP, Thermo Fisher Scientific, Bremen, Germany). The ^13^C concentration in the chambers decreased from 29.65 at% ^13^C (30 min after the second pulse) to 1.19 at% ^13^C (right before the end of the 4 h labeling period). The total CO_2_ concentration at the end of the labeling period was around 90 ppm. ^13^C measurements were conducted to indicate the successful labeling of the plant biomass. The plant material was analyzed using a mass spectrometer coupled with an elemental analyzer (QMS ESD 100, InProcess Instruments, Bremen, Germany).

The samples from the laboratory experiments were taken after 25 and 31 days of growth in the rhizoboxes (Vienna Scientific Instruments GmbH, Austria, PMMA, H × W × D, 23 × 10 × 3 cm) (Supplementary Table 1). The filling, fertilization, and planting were conducted as described for the soil column experiments (Lohse et al., 2020; Vetterlein et al., 2021).

For the verification of the MSI results, three technical replicates of disturbed BS and RS samples were taken from different positions of the rhizobox experiment. The RS was operationally defined as soil attached to the root for this comparison. The RS was manually separated from the roots by shaking the root. The BS was composed of soil that is not in the direct vicinity of the root. No embedding and cryosectioning were performed for the direct analysis *via* LDI-FT-ICR-MS to allow the comparison with the MSI workflow. The RS and BS were attached to adhesive tape for LDI measurement without additional sample preparation (Solihat et al., 2019).

### Preparation of the Samples for Mass Spectrometric Imaging

#### Embedding of Undisturbed Soil Samples

Embedding was done immediately after taking the samples from the rhizobox experiments. The field samples were stored at 5°C in the dark for transport from the field site until embedding at approximately 24 h after the sampling.

As for the embedding medium, a mixture of gelatin (5 weight percent) and CMC (2 weight percent) was prepared in MQW by slowly heating the mixture to 60°C and constant stirring by a magnetic stirrer until completely dissolved (Alfken et al., 2019). The infiltration of the undisturbed soil sample by the viscous embedding medium (max. 55°C) was only possible by applying a vacuum *via* a pump (110 mbar) for 15 min to the bottom of the moist undisturbed soil samples inside the metal cylinder to create suction while the warm medium was applied to the surface of the undisturbed soil sample.

Note that due to the high viscosity of the embedding medium, it does not infiltrate into the complete pore volume in the soil sample. Instead, only the major void volumes between the metal cylinder and soil core, or eventual fully connected macropores, are infiltrated when a vacuum is applied. Yet, this sufficiently improves the mechanical stability to enable subsequent cryosectioning and MSI.

After cooling down to room temperature, the embedded samples were stored in the freezer at −20°C (i.e., no quick shock freezing of the sample) until cryosectioning for no longer than 2 days. Longer storage time of the samples may lead to the disintegration of the sections during the cryosectioning presumably due to the loss of moisture.

#### Cryosectioning

The embedded and frozen undisturbed soil samples were removed from the metal cylinder after gentle warming of the outside. The undisturbed soil samples were attached to the specimen holder, completely coated with OCT compound before sectioning to provide additional stabilization, and placed in the cryomicrotome (cryostat Microm HM 560, Thermo Fischer Scientific, Waltham, United States, slide temperature: −15 to −19°C, blade temperature: −10 to −15°C). Sections of 80 to 100 μm thickness were cut from each undisturbed soil sample and thaw-mounted onto indium-tin oxide-coated (ITO) glass slides (Bruker Daltonik, Bremen, Germany). The soil sections were stored in a desiccator at 200 to 400 mbar at room temperature in the dark until measurement. Microscopic images of the soil sections were generated using a binocular microscope (M205 FA, Leica Microsystems GmbH, Wetzlar, Germany, field of view 17.3 mm × 13.0 mm) to preselect sections with a root and to identify the root surface. To further aid the detection of roots, fluorescence microscopy was used for some soil sections (UV emitted by a Leica EL6000 mercury metal halide lamp, 405 nm long pass filter). The fluorescence of the embedding medium can also be detected simultaneously. Regions around a root with a detectable fluorescence of the embedding medium should not be analyzed with the MSI method to avoid metabolite delocalization artifacts.

### LDI-FT-ICR-MS Measurements

#### Mass Spectrometric Imaging

All mass spectrometric measurements were performed with an FT-ICR-MS with a dynamically harmonized analyzer cell (solariX XR, Bruker Daltonics, Billerica, MA, United States) and a 12 T refrigerated actively shielded superconducting magnet (Bruker Biospin, Wissembourg, France). The mass spectrometer was controlled with ftmsControl 2.2.0 (Bruker Daltonics). Mass spectra were recorded in the mass window setting 147–1,000 *m/z* in magnitude mode (four megaword time domain, mass resolving power 550,000 at *m/z* 341) and reduced profile mode (97% data reduction). The FT-ICR-MS was run in the continuous accumulation of selected ions (CASI) mode (isolation *m/z* = 341.5, isolation width = 10 Da) to improve the signal-to-noise ratio (S/N) in the *m/z* window of the dihexose. As the target molecule, the mass of dihexose (C_12_H_22_O_11_, molecular formula of e.g., sucrose, maltose, lactose, and melibiose) was selected since these metabolites are commonly found in root exudates (Lugtenberg et al., 1999; Walter et al., 2003; Fan et al., 2012).

The external mass calibration was done with SRFA followed by an internal lock mass calibration using the *m/z* value 341.10894 [C_12_H_22_O_11_−H]^–^. A pulsed MALDI-source (smartbeam-II UV Laser; Bruker Daltonik, Nd:YAG, 355 nm, 2.14 eV, approximately 100–150 μJ/shoot at 100% laser power) was used in negative ionization mode. The number of single spectra on the analyzed soil sections was between 857–6,807 (Supplementary Table 1). Every single spectrum was generated with minimum laser focus (laser spot diameter approx. 20 μm) and 40 to 50 laser shots at a laser frequency of 400 to 500 Hz with 55–70% laser power with a raster width of 25 μm. For aligning the microscopic images with the respective position on a soil section before MSI, the software FlexImaging 4.0 (Bruker Daltonics) was used.

For the MSI of a mass window at a smaller center *m/z* value, the settings were changed as follows: CASI mode isolation *m/z* = 172.0, isolation width = 20 Da. For the internal lock mass calibration the *m/z* values 164.0717 [C_9_H_11_NO_2_−H]^–^, 173.0092 [C_6_H_6_O_6_−H]^–^, 179.0561 [C_6_H_12_O_6_−H]^–^, and 180.0666 [C_9_H_11_NO_3_−H]^–^ were used. The selected masses were previously reported as compounds found in root exudates (Fan et al., 2012).

For the MSI of the complete mass range (147 – 1000 *m/z*), no CASI mode was applied. Every single spectrum was generated by 30 laser shots at a laser frequency of 300 Hz with 35% laser power.

Experimental details on the LDI measurement of a sucrose standard can be found in the Supplementary Material to this article.

#### Bulk Soil and Rhizosphere Soil

The disturbed BS and RS samples on adhesive tapes were attached to an ITO slide. Thirty-two scans without CASI mode or data reduction were added for each spectrum. Every single scan was generated by 20 laser shots at a laser frequency of 200 Hz with 25% laser power.

The experimental details on the molecular formula assignment for disturbed soil samples can be found in the Supplementary Material to this article.

#### Blanks

Blank spectra for the gelatin/CMC mixture, the OCT compound, the ITO slide, and the MALDI steel target were generated to recognize possible contamination. Every single scan was generated by 50 laser shots at a laser frequency of 500 Hz with 70% laser power. Thirty-two scans with CASI mode *m/z* = 341.5, isolation width = 10 Da were co-added for each spectrum. No blank signal for the dihexose mass was observed for any of the tested blanks (Supplementary Figure 1).

### Data Processing for Mass Spectrometric Imaging

SCiLS Lab (Version 21a Core, Bruker Daltonik) in combination with the microscopic images were used to manually select regions of interest (ROI) around the root (Supplementary Table 1) and to define areas with an increasing distance to the root surface to visualize molecular gradients. The peak intensities were normalized to the root mean square (RMS) of the peak intensities in each spectrum, averaged for each region, exported, and further processed with R version 4.0.3 (R Core Team, 2020).

The Box-and-whisker plots denote the 75*^th^* and 25*^th^* percentiles (box), whiskers indicate 1.5 × interquartile range (IQR). For the statistical evaluation of the isotope ratios, one-sample *t*-tests were conducted (one-sided, non-paired, α = 0.05) to compare the mean isotope ratio (IR) for each distance <150 μm from the root and the root itself with the natural ^13^C_1_ abundance of the dihexose (0.1298). As an approximation of the measurement uncertainty of the IR, the relative IQR (IQR/median) of the IR for each distance <150 μm from the root and the root itself was calculated. As an estimation for the measurement uncertainty, the mean of these values was used (field sample: 10.8% and rhizobox: 10.0%).

The RMS normalized data were imported in METASPACE (Palmer et al., 2017) to find colocalized metabolites and to annotate possible metabolite structures by using the Database Chemical Entities of Biological Interest (ChEBI 2018-01) with a maximum 10% false discovery rate.

## Results

### Optimized Sampling and Sample Preparation

To preserve the small-scale gradients of the molecules in the undisturbed soil samples, small metal cylinders were used for sampling. These can be applied in laboratory and field experiments to take samples below the soil surface. The size for the undisturbed soil sample of up to 0.91 cm diameter and 1.0 cm height was chosen after we observed the disintegration of the sample and loss of soil for larger undisturbed soil samples especially during the embedding and cryosectioning step.

The sectioning of soil samples required embedding in a mixture of gelatin and CMC. Freeze-drying of the soil sample is not recommended as this may result in shrinkage, a brittle structure, and disintegration during the embedding step. Applying a vacuum (down to 110 mbar) to the bottom of the undisturbed soil sample created sufficient suction to infiltrate the soil with the medium. A simple placement of the undisturbed soil sample in a beaker containing the medium did not result in complete embedding and caused the disintegration of the section during the cryosectioning. After embedding, the soil samples were frozen at −20°C to limit the diffusion and microbial activity in the sample.

The stability of the soil sections was further improved by coating the outside of the embedded undisturbed soil sample with a layer of OCT compound before mounting it into the cryomicrotome. Sections of the loam <80 μm were too brittle and did not keep the soil structure around the root intact. Thicker sections (>100 μm) could not be cut reliably since the undisturbed soil sample would fall off the specimen holder due to the higher friction. To ensure that the storage and measurement in the vacuum did not affect the integrity of the sections, images were taken to evaluate the effect of reduced pressure. A small shrinkage of the soil section after storage in vacuum at 200 to 400 mbar for 80 min was observed (Supplementary Figures 2A,B). No further shrinkage in the vacuum (3 mbar) during the MSI measurement was observed (Supplementary Figures 2C,D).

### Direct Imaging of Root Exudates in Soil *via* Laser Desorption Ionization Ultra-High Resolution Mass Spectrometry

**FIGURE 1:**
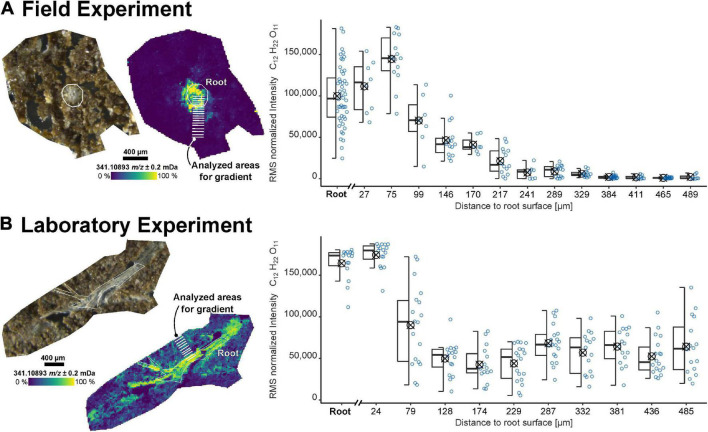
Imaging results for samples from **(A)** the field experiment (FP1, root cross-section) and **(B)** a laboratory experiment (RB1-b1, root longitudinal section). Optical images of the soil sections and ion images for the dihexose [C_12_H_22_O_11_−H]^–^ as detected by laser desorption ionization Fourier-transform ion cyclotron resonance mass spectrometry (LDI-FT-ICR-MSI) are shown on the left. The peak intensity of the dihexose signal [C_12_H_22_O_11_−H]^–^ versus maximum distance to the root surface for selected areas (each covering 7 to 19 spectra, each spectrum represented by one blue dot) is shown on the right. The root surface is highlighted in white and the areas used to construct the boxplots (symbol indicates arithmetic mean) are highlighted in the ion images.

The sample preparation workflow was applied to recover the spatially resolved information of the root exudates from both field (FP1) and laboratory experiments (RB1-b1). Dihexose (C_12_H_22_O_11_, molecular formula of e.g., sucrose, maltose, lactose, and melibiose) was selected as a target molecule for the MSI since these metabolites have been identified in root exudates (Lugtenberg et al., 1999; Walter et al., 2003; Fan et al., 2012). The highest intensity of the dihexose was detected in the root itself or within less than 100 μm from the root surface (Figure 1). While the intensity decreased gently over 500 μm for the sample taken from the field experiment, the laboratory sample showed a sharp drop within approximately 100 μm from the root surface.

Due to the heterogeneous distribution of signal intensity in the sections, the influence of the shape and size of the ROI on the mean intensities of the dihexose was tested with all soil sections (Supplementary Figures 3, 4 and Supplementary Table 1). The intensity gradient of the dihexose could be recovered in all samples, independent of the size and shape of ROI (small ROI: Figure 1, large ROI: Supplementary Figure 5).

**FIGURE 2:**
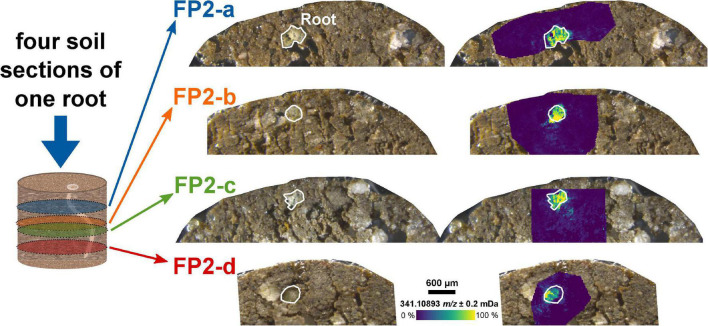
Molecular gradient of the dihexose along the root axis. Optical images of the soil sections and overlay with the ion image for the dihexose signal [C_12_H_22_O_11_−H]^–^ as detected by LDI-FT-ICR-MSI for the same root in different depths of an undisturbed soil sample from the field experiment. The root surface is highlighted in white.

The analysis of the two-dimensional (2D) sections from different depths of the soil sample can be used to reconstruct three-dimensional (3D) distributions of root exudates along the root axis. Four sections of the same root – a few 100 μm apart – showed a comparable intensity decrease for the dihexose mass with increasing distance to the root (FP2-a to FP2-d, Figure 2 and Supplementary Figure 5A).

**FIGURE 3:**
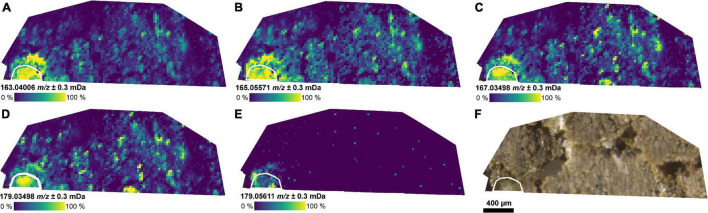
Distribution of small metabolites in the rhizosphere. **(A–E)** Ion images for metabolites co-localized with the position of the root as detected by LDI-FT-ICR-MSI of an undisturbed soil sample from the field experiment (FP2-d) and annotated *via* METASPACE: **(A)** [C_9_H_8_O_3_−H]^–^ (*m/z* 163.0401, e.g., coumaric acid), **(B)** [C_9_H_10_O_3_−H]^–^ (*m/z* 165.0557), **(C)** [C_8_H_8_O_4_−H]^–^ (*m/z* 167.0350, e.g., vanillic acid), **(D)** [C_9_H_8_O_4_−H]^–^ (*m/z* 179.0350, e.g., caffeic acid), **(E)** [C_6_H_12_O_6_−H]^–^ (*m/z* 179.0561, e.g., glucose), and **(F)** optical image of the soil section. The root surface is highlighted in white.

The masses of coumaric acid, vanillic acid, caffeic acid (aromatic carboxylic acids), and glucose could be detected in the root and the rhizosphere by selecting a CASI window with a smaller center *m/z* value (*m/z* 172 ± 10 Da, FP2-d, Figure 3). These compounds have been previously reported in root exudates (Supplementary Table 2). The detection of aromatic compounds is expected because of their selective better ionization with LDI compared with compounds without an aromatic system (Karas et al., 1985). A second LDI measurement of the same section (FP2-d), then, with the higher center *m/z* value (*m/z* 341.5 ± 5 Da) also revealed the mass of the dihexose around the same part of the root (Figure 2 and Supplementary Figure 3E).

We, therefore, tested the feasibility to conduct repeated LDI measurements of the same region (RB2) to expand the accessible mass range *via* selecting different CASI mass windows. Conducting two subsequent measurements of the same region did not alter the mean intensity of the dihexose. Even after the third and fourth measurements of the same section, 60 and 33% of the initial intensity could still be observed (Supplementary Figure 6) with, however, blurred intensity distributions. After four repeated measurements, the measurement region was charred and the section of the root within this region lost its UV-activity (Supplementary Figures 2E,F).

In sample FP1, mass peaks within a 10 Da mass range (*m/z* 341.5 ± 5 Da) were annotated *via* METASPACE (Palmer et al., 2017) and 17 metabolites from the ChEBI-Database were found. Seven of these showed a strong localization in the root, among them the dihexose (C_12_H_22_O_11_). The co-located metabolites were C_18_H_18_O_7_, C_19_H_20_O_6_, C_19_H_18_O_6_, and C_19_H_16_O_6_ (co-localization value with the C_12_H_22_O_11_: 0.59 – 0.76), but their identity as plant metabolites was not further evaluated (Supplementary Table 3). Additionally, C_6_H_12_O_6_ and C_6_H_10_O_5_ were detected in the spectra (*m/z* 341.5 ± 5 Da), pointing to the fragmentation of saccharides due to the cleavage of the glycosidic bond and additional loss of water. This fragmentation pattern was confirmed with an LDI-CID-FT-ICR-MS/MS (CID: collision-induced dissociation) experiment of a sucrose standard (Supplementary Figures 7A,B). Without the application of CASI-isolation and collision voltage, only a low intensity of fragmentation was observed, confirming a soft ionization when using LDI (Supplementary Figure 7C).

As an alternative approach to using selected mass windows, a full scan MSI measurement was tested with one section (FP2-d), revealing a high number of signals (Supplementary Figure 8). Only two metabolites could be annotated for the full scan MSI measurement *via* METASPACE with, however, no spatial correlation to the position of the root (Supplementary Figure 9).

### Validation of the Mass Spectrometric Imaging Results

To verify the imaging results with a conventional, destructive approach, disturbed BS and RS samples from a laboratory experiment were generated and analyzed by LDI-FT-ICR-MS. Next to a common background of complex soil OM, molecular formulas unique for BS and RS were detected (Supplementary Figure 10A), resulting in a higher mean O/C-ratio and a lower mean H/C-ratio in the RS sample as compared with the BS (Supplementary Table 4). In particular, this experiment confirmed the enrichment of putative sugars with high O/C and H/C ratios in the RS (Supplementary Figure 10B).

**FIGURE 4:**
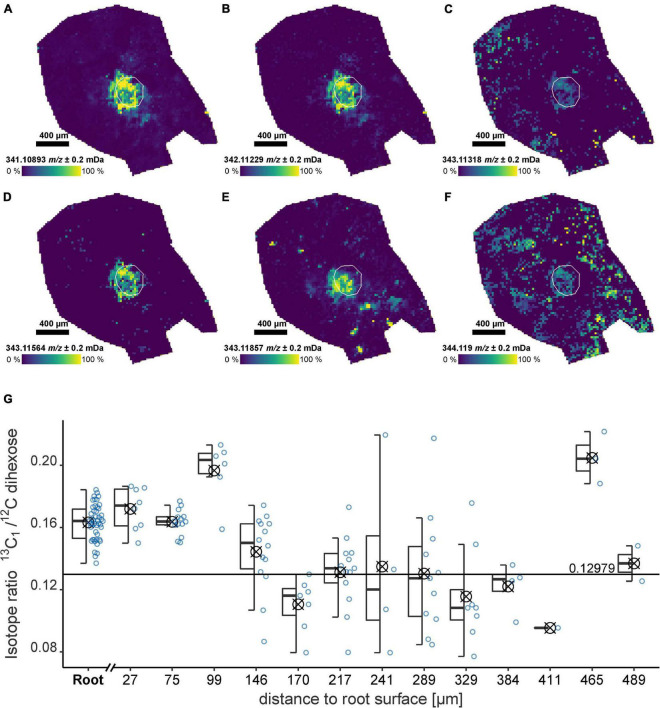
Dihexose Isotopologue and isotope ratio for an undisturbed soil sample from the field experiment (FP1) after ^13^C pulse labeling as detected by LDI-FT-ICR-MSI. **(A–F)** Ion images for Isotopologue signals of the dihexose [C_12_H_22_O_11_−H]^–^. The root surface is highlighted in white. **(A)** Monoisotopic signal [^12^C_12_^1^H_22_^16^O_11_−H]^–^, **(B)** ^13^C_1_, **(C)** ^18^O_1_**, (D)** ^13^C_2_**, (E)** ^13^C_1_^2^H_1_**, (F)** ^13^C_3_, and **(G)** Isotope ratio for regions versus maximum distance to the root surface considering the ^13^C_1_/^12^C ratio of the dihexose [C_12_H_22_O_11_−H]^–^ for all laser spots with detectable ^13^C_1_-Signal (natural abundance: 0.1298). The isotope ratio for each laser spot is shown as a blue circle. The areas used to construct the boxplots (symbol indicates arithmetic mean) are highlighted in the ion image in Figure 1A.

The ultra-high resolution of the FT-ICR-MS allows the detection of multiple Isotopologue signals simultaneously. For the dihexose, the ^13^C_1_, ^18^O_1_, ^13^C_2_, ^13^C_1_^2^H_1_, and ^13^C_3_-Isotopologue were detected localized in the root (Figures 4A–F, exact masses Supplementary Table 5). For both, the field and laboratory experiment, the intensity of the monoisotopic signal and the ^13^C_1_-isotopologue followed a linear trend. The slope *m* of the regression can be used to calculate the ^13^C_1_/^12^C isotope ratio (IR). The sample from the field experiment showed a higher slope (FP1, *m* = 0.17), indicating a possible ^13^C enrichment compared with the non-labeled laboratory experiment (RB1-b1, *m* = 0.14) and a sucrose standard (*m* = 0.13, Supplementary Figure 11).

The IR for a sample from the field experiment (FP1) where a ^13^C label was applied decreased with increasing distance to the root. In the root and below 150 μm from the root surface, the IR was significantly (*p* < 0.05) above the natural abundance (0.1298). The ^13^C uptake by the plant associated with sample FP1 was confirmed by IR mass spectrometry. ^13^C enrichment was detected for the shoot (2.53 ± 0.014 at% ^13^C) and the youngest unfolded leaf (1.44 ± 0.004 at% ^13^C). The mean natural abundance at BBCH 19 for the unlabeled plants was 1.078 ± 0.001 at% ^13^C (*n* = 24).

Due to the overall decrease in signal intensity with increasing distance to the root (Figure 1A), the coefficient of variation of the IR for each area increased (Figure 4G). Using the relative IQR, we estimated a measurement uncertainty for the spatially and molecular resolved IR of approximately 10%. For the non-labeled laboratory sample (RB1-b1), only inside the root, a significantly higher IR could be detected (*p* = 0.04), while in the rhizosphere (<150 μm from the root surface) the IR reflects the natural ^13^C_1_ abundance (Supplementary Figure 12).

## Discussion

### Suitability of the Sampling and Sample Preparation Workflow

A sample preparation method was developed allowing the spatially resolved analysis of organic molecules at the root-soil interface using LDI coupled with ultra-high resolution mass spectrometry. The primary focus of the sample preparation was to generate thin and stable soil sections. Crucial aspects of the sampling and sample preparation workflow are discussed in the following.

Non-embedded, undisturbed soil samples are not stable during sample preparation and can easily disintegrate during cryosectioning. Freeze-drying of the soil samples resulted in a major volume reduction and a brittle structure of the soil. Conventional resin-embedding requires chemical fixation and the removal of water before the embedding (Mueller et al., 2013; Bandara et al., 2021) and is prone to extract or delocalize small molecules from the soil. Gelatin was previously used for the stabilization of soil samples for microscopic examination of soil structure (Anderson, 1978), and a mixture of gelatin and CMC was recently used for sediment samples (Alfken et al., 2019; Wörmer et al., 2019).

In our workflow, the embedding medium was directly applied to the field moist sample. However, applying the gelatin/CMC mixture onto the top of the undisturbed soil sample resulted in the insufficient embedding/stabilization of the sample, and the obtained sections were brittle and could not be mounted intact onto a glass slide. Therefore, a vacuum was applied to the bottom of the undisturbed soil sample. This supported the penetration of the embedding medium into the soil sample. It should be noted that already a partial filling of the pore volume (as shown in the microscopic images, Supplementary Figure 13) by the embedding medium considerably improved the stability of the sample for subsequent cryosectioning. To minimize the microbial degradation and the diffusion of metabolites, it is recommended to conduct the embedding immediately after sampling. If applicable, non-destructive imaging techniques such as X-ray CT (Gao et al., 2019) could help to pre-select undisturbed soil samples.

The thickness of a section often has a strong effect on the sensitivity of the MSI detection (Alfken et al., 2019). However, for our workflow, the section thickness is limited by the stability of the sections, and sections thinner than 80 μm and thicker than 100 μm tended to be less stable. It should be mentioned that the optimal thickness depends on the substrate texture. For substrates with a higher proportion of sand (i.e., when most of the single particles are larger than 200 μm), the section thickness must be adapted accordingly. To aid identification of ROI containing a root, non-destructive imaging techniques such as optical or fluorescence microscopy can be applied to the sections before subsequent MSI analysis.

### Capabilities and Limits of the Laser Desorption Ionization Method

As an ionization method, LDI was used. The soil mineral matrix and the aromatic compounds in the root and SOM absorb and distribute the laser energy, even allowing for multiple measurements of the same region (Supplementary Figure 6). A MALDI matrix is often used in MSI and has also been applied for indirect rhizosphere gradients (Veličković et al., 2020b). However, the application of a matrix is an additional sample preparation step that may lead to the diffusion of target molecules on the surface of the section and could also contribute to additional background signals, limiting the ion abundance and hence detection limit of the relevant metabolites.

To achieve the highest sensitivity for observing the gradients of metabolites in the soil despite the SOM background (Supplementary Figure 8), we propose to limit the mass window to a maximum of 20 Da (CASI mode). Although this reduces the number of target molecules detectable in one imaging run, it ensures the sensitive detection of metabolites in soil with high spectral quality.

Targeting other metabolites outside the selected mass window may require multiple MSI measurements with accordingly increased measurement time. E.g., the distribution of [C_9_H_8_O_3_−H]^–^ (*m/z* 163.0401, e.g., coumaric acid) and the dihexose [C_12_H_22_O_11_−H]^–^ (*m/z* 341.1089, e.g., sucrose) could not be determined in one measurement, but it was possible with two successive measurements from the same section (Supplementary Figure 3E and Figures 2, 3). The inclusion of further mass windows may be limited by the loss of intensity after two consecutive LDI measurements (Supplementary Figure 6).

Without the application of the CASI mode, gradients of metabolites could not be detected around the root (Supplementary Figure 9). However, a large number of signals in the full mass spectrum (Supplementary Figure 8), point to the ionization of SOM by LDI (Solihat et al., 2019), which could be used to analyze the changes in the SOM composition due to rhizosphere processes. The observed chemical complexity further highlights the need for ultra-high mass resolution, as offered by FT-ICR-MS. The further extension of the MSI method to a larger mass range may allow for a comprehensive non-targeted metabolite analysis in the rhizosphere.

Here, we used disturbed soil samples (i.e., *via* mechanical separation into BS and RS) to screen (no embedding and cryosectioning, Solihat et al., 2019) for target molecules used in subsequent imaging runs (Supplementary Figure 10). Using the LDI measurements of BS and RS samples from a laboratory experiment, the enrichment of molecular formulas of putative sugars with high O/C-ratio was observed in the RS (Supplementary Figure 10B).

The overall MSI workflow was tailored to prevent the delocalization of metabolites. By having short storage times either at low temperature or in a vacuum, and just the necessary sample preparation steps (i.e., no MALDI-matrix application), artifacts were minimized. Since the embedding medium does not infiltrate the complete pore volume (as detected *via* fluorescence microscopy, see Supplementary Figure 13) and was used sparingly, the bias of the embedding process can be minimized. In any case, we could find the highest intensity close to the root surface and always a good correlation of the position of the root with the metabolite signals, pointing to little or no bias by the sample preparation.

### Benefits of Laser Desorption Ionization-Mass Spectrometric Imaging for Rhizosphere Research

The spatial resolution of LDI-FT-ICR-MSI (25 μm) is sufficient to reveal the distribution of individual plant metabolites in the soil, which cannot be achieved using bulk analysis. A strong decrease in the intensity of the dihexose with increasing distance to the root up to 150 μm could be detected (Figure 1 and Supplementary Figure 5). The low dihexose intensity in regions furthest away from the root indicates a low level of blank contribution by the sample preparation workflow (see also Supplementary Figure 1). The non-linear decrease in intensity can be caused either by microbial utilization (Fischer et al., 2010) or by sorption to minerals or organic matter in the soil (Kaiser and Guggenberger, 2003) but is also expected from diffusive gradients around the roots (Raynaud, 2010; Vetterlein et al., 2020; Landl et al., 2021).

The less pronounced gradient in the laboratory experiments compared with the field experiment may be explained by the higher water content and hence, increased diffusion during the laboratory experiments (Raynaud, 2010; Holz et al., 2018b). Additionally, the higher root length density and different root age in the laboratory compared with the field experiment could lead to changing carbon input by the roots into the soil (Vetterlein et al., 2021). Molecular gradients in a soil section could also be affected by the proximity of the underlying neighboring roots below the 2D-plane analyzed by MSI, which can be identified by X-ray CT. The shape of the observed molecular gradient may also depend on the orientation of the 2D plane analyzed by MSI concerning the root axis (i.e., cross-section or longitudinal section).

As shown in Figure 3, several metabolites could be identified in and around the root. All the compounds assigned to the detected exact masses have been described previously as root exudate components (Supplementary Table 2). However, the origin of the compounds cannot be specified – potential microbial metabolites could contribute to the observed gradient. Structural identification of the detected masses may be achieved by orthogonal analysis using chromatographic techniques coupled with mass spectrometry (van Dam and Bouwmeester, 2016). Differences in the distribution of certain molecular species may, again, depend on differences in the initial concentration or selective adsorption to minerals.

The distribution and decomposition of organic compounds released by the roots are often determined by using isotopic labels such as ^14^C, ^15^N, or ^13^C (Pausch and Kuzyakov, 2012; Pausch et al., 2013; Vidal et al., 2018). Depending on the experimental setup used, the detectable size of the rhizosphere can vary from a few hundred μm (Holz et al., 2019; Bilyera et al., 2021) up to several mm (Schenck zu Schweinsberg-Mickan et al., 2010; Holz et al., 2018b). The maximum distance from the root where the dihexose could be detected was well below 1 mm. Considering that a single molecular formula was analyzed, the corresponding lower sensitivity as compared with measuring a bulk isotope label could be the reason for the apparent smaller extent of the rhizosphere. Bilyera et al., 2021 reported an observable rhizosphere extent of 120 μm and 880 μm for a mature wild type maize root and the root tip region, respectively based on ^14^C imaging, which correlates well with our results (highest intensity of the dihexose within less than 100 μm from the root surface).

Several Isotopologues (^13^C, ^18^O, ^2^H) of the dihexose were detected in the root (Figure 4), indicating a possible application of the presented workflow for the detection of stable isotope-labeled metabolites. It should be mentioned that even without the application of a ^13^C pulse labeling, the natural isotope abundance can be detected (Supplementary Figure 12), highlighting the versatility of the LDI-FT-ICR-MS workflow. Although ultra-high resolution mass spectrometry provides information about the accurate mass of Isotopologues, the precision and accuracy of the IR (as determined from the peak intensities) depends on the absolute magnitude of the detected signal as described for Orbitrap-MS (Khodjaniyazova et al., 2018) and FT-ICR-MS (Cao et al., 2021).

For our LDI-FT-ICR-MS workflow, the ion abundances and accuracy of the ^13^C_1_/^12^C IR were affected by the concentration of the analyte and the laser power (Supplementary Figures 14, 15). Such information is crucial to calculate thresholds for a reliable determination of the ^13^C_1_/^12^C IR. In the sample from the field experiment for which ^13^C labeling was applied, a dihexose labeling could be detected up to a distance of 150 μm from the root surface, based on the significantly higher IR values (Figure 4) pointing to the enrichment of ^13^C in the rhizosphere. This was confirmed by IR-MS, showing ^13^C enrichment in the plant biomass and a strong depletion of ^13^CO_2_ in the atmosphere during the labeling period. We conclude that combining our presented MSI workflow with stable isotopic labeling could therefore provide even more specific insight into *in situ* root exudation and for the characterization of metabolite pools (Arrivault et al., 2017).

## Conclusion

We established a workflow to directly analyze metabolite gradients within a few hundred μm from the root surface in the rhizosphere *via* LDI-FT-ICR-MSI. For the first time, the method enabled us to study chemical gradients with a high spatial resolution (25 μm) directly in the soil. The visualization of multiple metabolites is possible and stable isotope-labeled compounds can be detected in the rhizosphere. Visualizing the root and soil structure non-invasively *via* X-ray CT, magnetic resonance imaging, or positron emission tomography in an undisturbed soil sample before the embedding would enable a guided sampling approach to analyze molecular distributions at certain parts of the root. Moreover, the direct molecular imaging of the rhizosphere *via* LDI-FT-ICR-MS could be correlated with elemental imaging. Sampling diffusive gradients in thin films and measurement *via* laser ablation – inductively coupled plasma – mass spectrometry (DGT LA-ICP-MS) may allow for additional insight into the chemical gradients in the rhizosphere (Wagner et al., 2020).

Interpolating between subsequent sections along the root axis could be used to reconstruct 3D plant metabolite distributions in the rhizosphere with high spatial resolution. This would allow relating changes in the metabolite profile along the root axis (Jaeger et al., 1999; Walter et al., 2003; Holz et al., 2018a) to root age or ontogeny. Combining sample preparation and ultra-high resolution measurement on such a small scale will help to unravel the interplay between roots, microbes, and soil and to understand the fate of root exudates in the rhizosphere.

## Data Availability Statement

The datasets presented in this study can be found in online repositories. The name of the repository and link can be found below: https://metaspace2020.eu/project/Lohse_2021_imaging_metabolites_rhizosphere.

## Author Contributions

ML developed the sample preparation and MSI method, conducted the data analysis, and compiled the manuscript. RH supported the sample preparation and MSI method development. EL conducted the ^13^C labeling in the field experiment. DV acquired funding and managed the project. TR supported the method development. OL acquired funding, managed and supervised the project, and supported the method development and the data analysis. All authors contributed to the article and approved the submitted version.

## Conflict of Interest

The authors declare that the research was conducted in the absence of any commercial or financial relationships that could be construed as a potential conflict of interest.

## Publisher’s Note

All claims expressed in this article are solely those of the authors and do not necessarily represent those of their affiliated organizations, or those of the publisher, the editors and the reviewers. Any product that may be evaluated in this article, or claim that may be made by its manufacturer, is not guaranteed or endorsed by the publisher.
