# Supplementary material for: Direct Imaging of Plant Metabolites in the Rhizosphere Using Laser Desorption Ionization Ultra-High Resolution Mass Spectrometry
Source: Front Plant Sci. 2021 Dec 3;12:753812. doi: 10.3389/fpls.2021.753812 (PMC8678481; doi:10.3389/fpls.2021.753812)
Supplement: Supplementary file 1 [file Data_Sheet_1.pdf]

Supplementary Material

Direct Imaging of Plant Metabolites in the Rhizosphere Using Laser Desorption Ionization Ultra-High Resolution Mass Spectrometry

Martin Lohse*[1]; Rebecca Haag[1,2]; Eva Lippold[3]; Doris Vetterlein[3,4]; Thorsten Reemtsma[1,5]; Oliver J. Lechtenfeld*[1,6]

[1] Department of Analytical Chemistry, Helmholtz Centre for Environmental Research – UFZ, Leipzig, Germany
[2] Ansbach University of Applied Sciences, Ansbach, Germany
[3] Department of Soil System Science, Helmholtz Centre for Environmental Research – UFZ, Halle/Saale, Germany
[4] Soil Science, Martin Luther University Halle-Wittenberg, Halle/Saale, Germany
[5] Institute of Analytical Chemistry, University of Leipzig, Leipzig, Germany
[6] ProVIS – Centre for Chemical Microscopy, Helmholtz Centre for Environmental Research – UFZ, Leipzig, Germany

***Correspondence:** Corresponding Authors

martin.lohse@ufz.de

oliver.lechtenfeld@ufz.de

Contents

Experimental details on the LDI measurement of a sucrose standard and the molecular formula assignment for disturbed soil samples 3
LDI measurement of a sucrose standard 3
Molecular formula assignment for disturbed soil samples 3
Additional Tables and Figures 4
Table S1: Sampling and measurement metadata. 4
Figure S1: LDI-FT-ICR-MS mass spectra of the blanks. 5
Figure S2: Soil section during sample preparation and measurement. 6
Figure S3: Imaging results for undisturbed soil samples from the field experiment. 7
Figure S4: Imaging results for undisturbed soil samples from the laboratory experiment. 8
Figure S5: Decrease of the peak intensity of the dihexose signal $[C_{12}H_{22}O_{11}-H]^{-}$ as detected by LDI-FT-ICR-MSI in regions of interest with increasing maximum distance to the root surface 9
Table S2: Molecular formula annotation for the MSI data of soil section FP2-d (*m/z* 172 ± 10 Da). 10
Figure S6: Repeated measurement of the same region on a soil section. 11
Table S3: Molecular formula annotation for the MSI data of soil section FP1 (*m/z* 341.5 ± 5 Da). 12
Figure S7: LDI-CID-FT-ICR-MS/MS experiment of the sucrose standard. 13
Figure S8: Zoom into an LDI-FT-ICR-MS mass spectrum averaged for the whole analyzed region of a soil section (FP2-d) without applying the CASI mode (full scan spectrum). 14
Figure S9: Localization of plant metabolites without applying the CASI mode is not possible. A) 15
Figure S10: Comparison of the intensity ratio for disturbed bulk- and rhizosphere soil samples using LDI-FT-ICR-MS. 16
Table S4: Aggregated molecular parameters for disturbed bulk - and rhizosphere soil samples after isolation of the soil from the root and direct measurement of soil without embedding via LDI-FT-ICR-MS. 17
Table S5: Exact masses and natural abundance for the isotopologues of the ion formula of a dihexose $[C_{12}H_{22}O_{11} - H]^{-}$ 18
Figure S11: Correlation plots for the $^{13}C_1$ and ^{12}C intensity of the dihexose signal $[C_{12}H_{22}O_{11}-H]^{-}$ for the LDI-FT-ICR-MSI. 19
Figure S12: Isotope ratio for an undisturbed soil sample from a laboratory experiment (RB1-b1) as detected by LDI-FT-ICR-MSI. 20
Figure S13: Optical (left) and UV-fluorescence images (right) of soil sections and the embedding medium. 21
Figure S14: Dependency of the $^{13}C_1/^{12}C$ ratio of sucrose $[C_{12}H_{22}O_{11}-H]^{-}$ on the ^{12}C-intensity. 22
Figure S15: $^{13}C_1/^{12}C$ ratio of sucrose $[C_{12}H_{22}O_{11}-H]^{-}$ for different measurement conditions. 23
Supplementary References 24

Experimental details on the LDI measurement of a sucrose standard and the molecular formula assignment for disturbed soil samples

LDI measurement of a sucrose standard

Three concentration levels of sucrose in MQW (0.1, 10, 1000 ppm) were spotted on a MALDI steel target (2 × 7.5 µL for each spot). 39 single scans with CASI mode *m/z* = 341.5, isolation width = 10 Da were recorded with 40 laser shots at a laser frequency of 400 Hz with 70% and 50% laser power for each concentration level. Additional measurements for the 0.1 ppm sucrose standard were performed with 30% laser power and for a 1000 ppm sucrose standard with 70% laser power without CASI-isolation (full scan) (Figure S11, Figure S14, Figure S15). An LDI-CID-FT-ICR-MS/MS (CID: collision-induced dissociation) experiment was performed using the 10 ppm sucrose standard to identify possible fragments. For one spectrum 32 scans were co-added with 15 V collision voltage (Figure S7).

Molecular formula assignment for disturbed soil samples

For the BS- and RS samples, internal recalibration of averaged spectra was done with a list of masses commonly present in natural organic matter (*m/z* 149 - 719, *n* = 253, linear calibration function). RMS error of the calibration masses was below 0.2 ppm. Peaks were considered detected if the S/N was greater than four. Raw spectra were processed with Compass DataAnalysis 5.0 (Bruker Daltonics). Molecular formulas were assigned to peaks in the range 148–1000 *m/z* allowing for elemental compositions $C_{1–80}$ $H_{1–198}$ $O_{0–40}$ $N_{0–2}$ $S_{0–1}$ with an error range of ±0.5 ppm (Koch et al., 2007; Lechtenfeld et al., 2014). Briefly, the following rules were applied: 0 ≤ H/C ≤ 3.0, 0 ≤ O/C ≤ 1.2, 0 ≤ N/C ≤ 1.5, 0 ≤ DBE ≤ 50 (double bound equivalent, DBE = 1 + 1/2 (2C − H + N) (Koch et al., 2014), −10 ≤ DBE-O ≤ 20 (Herzsprung et al., 2014), and element probability rules proposed by Kind and Fiehn (Kind and Fiehn, 2007). Isotope formulas (^{13}C, ^{34}S) were used for quality control but removed from the data set as they represent duplicate chemical information. The mass error range in the final data set was limited to the 5th–95th percentile of errors of CHO formulas in the initial data set (approx. ± 0.431 ppm). Molecular descriptors of peak intensity weighted average (wa) and mean values were calculated (Table S4) for H/C, O/C, N/C, S/C, N/S, DBE, DBE-O, and AI (aromaticity index: (1 + C - O - S - 0.5 · (H + N))/(C - O - S - N)) (Herzsprung et al., 2014). Relative peak intensities (RI) were calculated based on the summed intensities of all assigned monoisotopic peaks (TIC) in each sample. Van Krevelen diagrams for RS samples and BS samples were used to depict differences in relative intensities (ΔRI) for each molecular formula: $\Delta RI = RI_{RS}/(RI_{BS} + RI_{RS})$ (Figure S10).

Additional Tables and Figures

Table S1: Sampling and measurement metadata. Name of the soil section, type of experiment, age of the plants sampled, and sampling depth from the soil surface. The number of scans for the regions of interest (ROI) and the respective area are given for the total measured region and the smaller ROI drawn to determine rhizosphere gradients (ROI 1 to ROI 4 or ROI 5) by LDI-FT-ICR-MSI.

Soil section	Experiment	Age of Plant (days after planting)	Sampling depth (cm)	ROI - Number of Scans	ROI - area (mm^2)
FP1	field plot	64	12	4749, 161, 273, 468, 538	2.97, 0.10, 0.17, 0.29, 0.34
FP2-a	field plot		21	3478, 259, 371, 543, 416	2.17, 0.16, 0.23, 0.34, 0.26
FP2-b	field plot			4368, 202, 170, 289, 456, 789	2.73, 0.13, 0.11, 0.18, 0.29, 0.49
FP2-c	field plot			4039, 202, 220, 378, 547, 973	2.52, 0.13, 0.14, 0.24, 0.34, 0.61
FP2-d	field plot			2025, 232, 301, 451, 527	1.27, 0.15, 0.19, 0.28, 0.33
RB1-a	laboratory (rhizobox)	25	16.5	5955, 173, 587, 528, 678	3.72, 0.11, 0.37, 0.33, 0.42
RB1-b1	laboratory (rhizobox)		7.5	6358, 541, 267, 300, 291	3.97, 0.34, 0.17, 0.19, 0.18
RB1-b2	laboratory (rhizobox)			6807, 477, 702, 956, 589	4.25, 0.30, 0.44, 0.60, 0.37
RB1-c1	laboratory (rhizobox)		9	5563, 140, 196, 163, 141	3.48, 0.09, 0.12, 0.10, 0.09
RB1-c2	laboratory (rhizobox)			4538, 376, 281, 292, 254, 313	2.84, 0.24, 0.18, 0.18, 0.16, 0.20
RB2	laboratory (rhizobox)	31	10.5	857*	0.54*

* Value for the first measurement replicate.

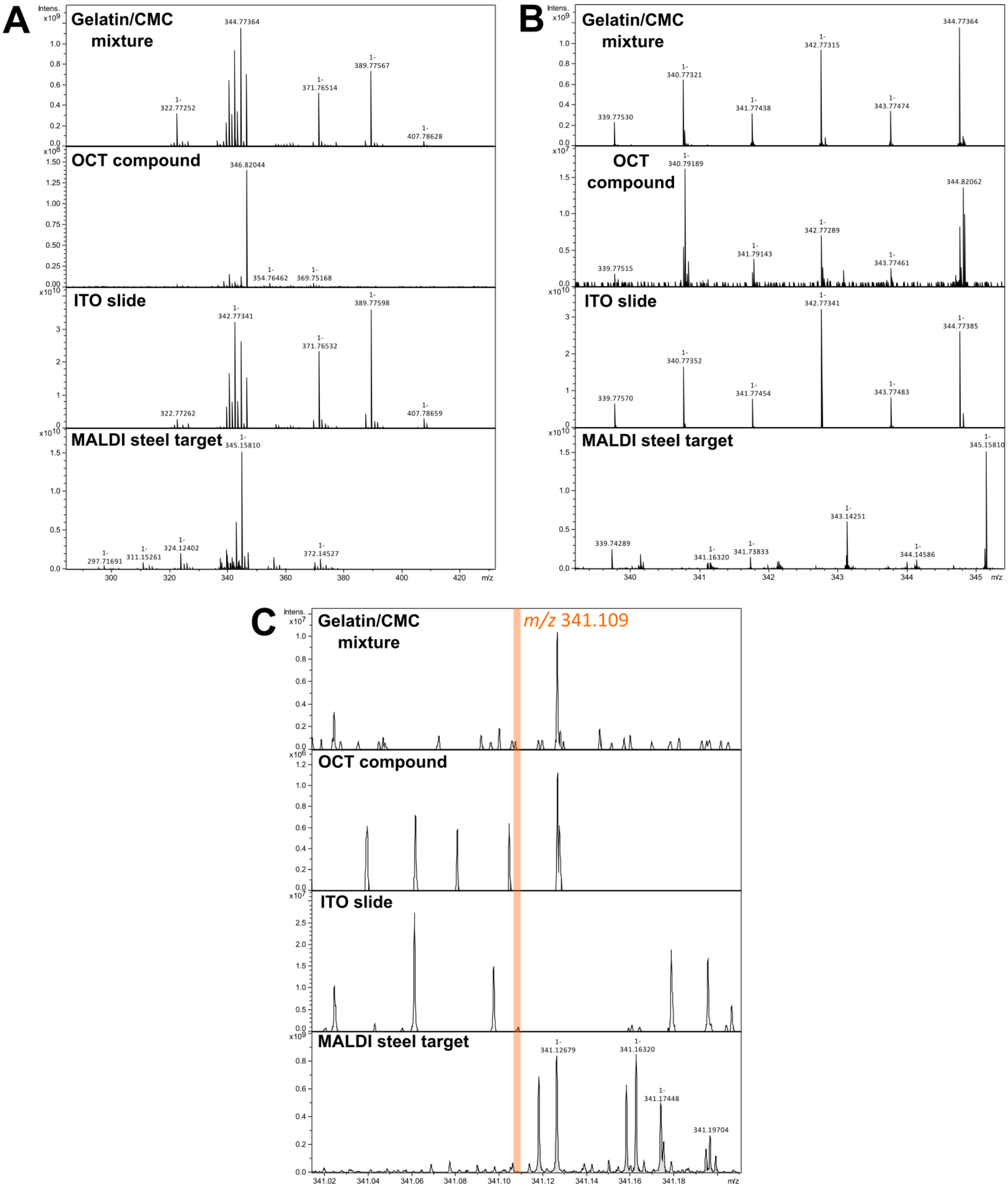

Figure S1: LDI-FT-ICR-MS mass spectra of the blanks. A/B) Blank spectra of 32 coadded spectra of the gelatin/ CMC mixture, the OCT compound, the ITO-slide, and the MALDI steel target (top to bottom) C) Zoom into the nominal mass of the dihexose ($[C_{12}H_{22}O_{11}-H]^-$, *m/z* 341.1089, highlighted) a blank signal above the noise level of this mass could not be detected.

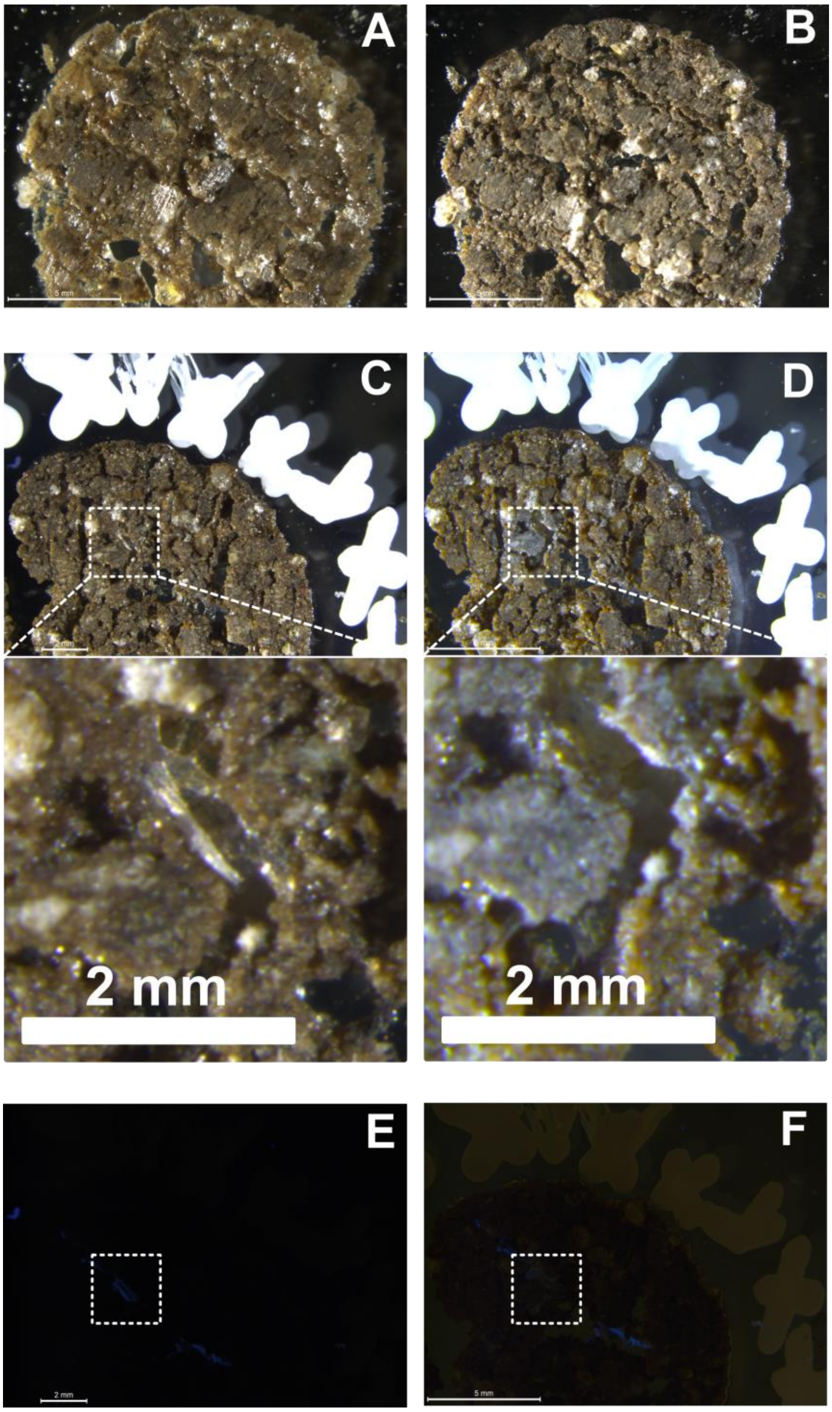

Figure S2: Soil section during sample preparation and measurement. A) soil section before and B) after storage in a vacuum (200 – 400 mbar) for 80 min before MSI. C) Optical image of the measurement region of soil section RB2 before MSI and D) after four times measurement of the same region. E) Optical image with UV excitation of the measurement region of soil section RB2 before MSI and F) after four times measurement of the same region. The area with loss of fluorescence is highlighted.

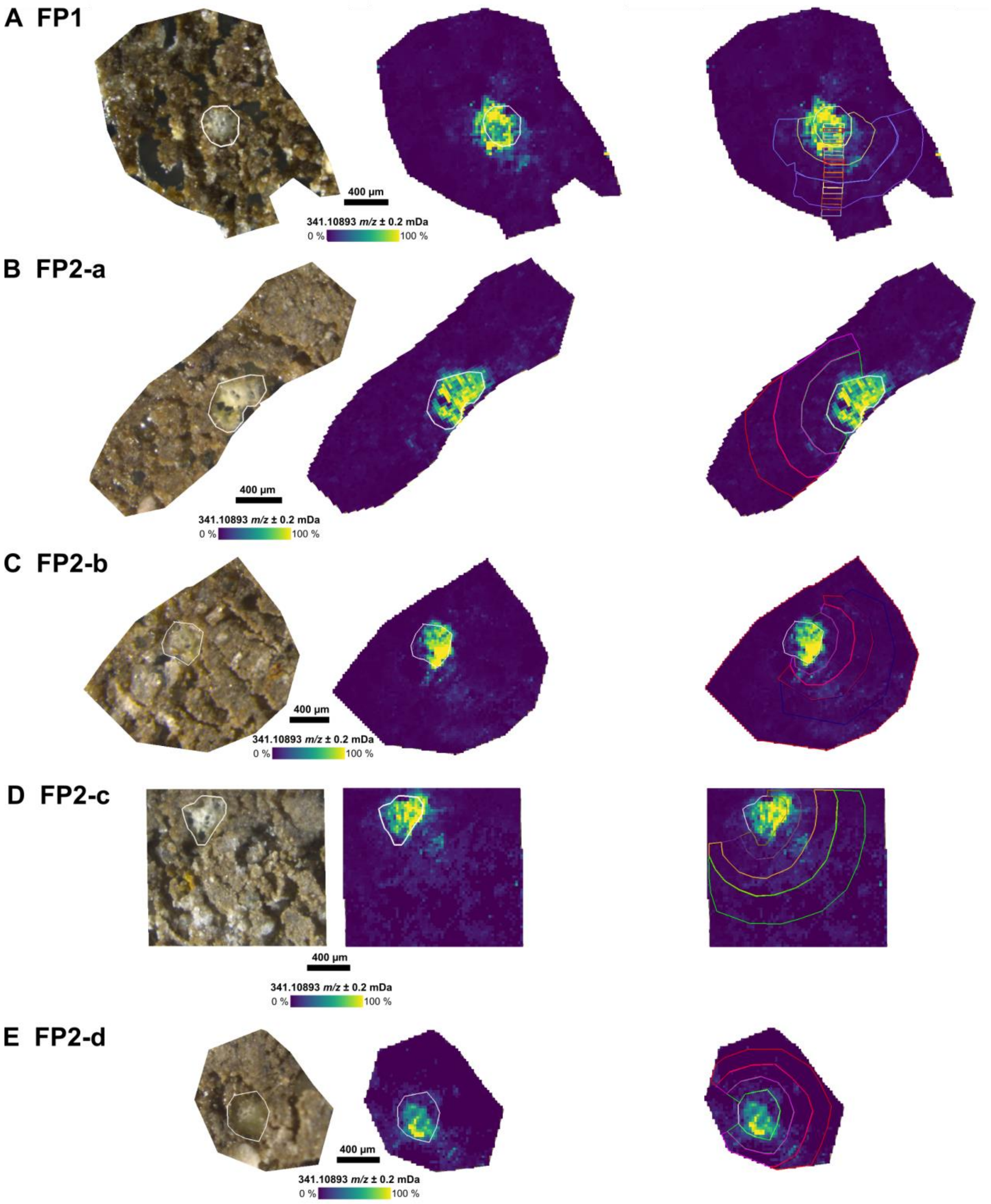

Figure S3: Imaging results for undisturbed soil samples from the field experiment. Optical image of the soil section and ion image for the dihexose signal $[C_{12}H_{22}O_{11}{-}H]^-$ as detected by LDI-FT-ICR-MSI the root surface is highlighted in white. Right: Ion image for the dihexose signal $[C_{12}H_{22}O_{11}{-}H]^-$ with the analyzed regions of interest for the rhizosphere gradients (ROI 1 is the root, ROI 2 to ROI 4 or 5 are regions with increasing distance to the root surface).

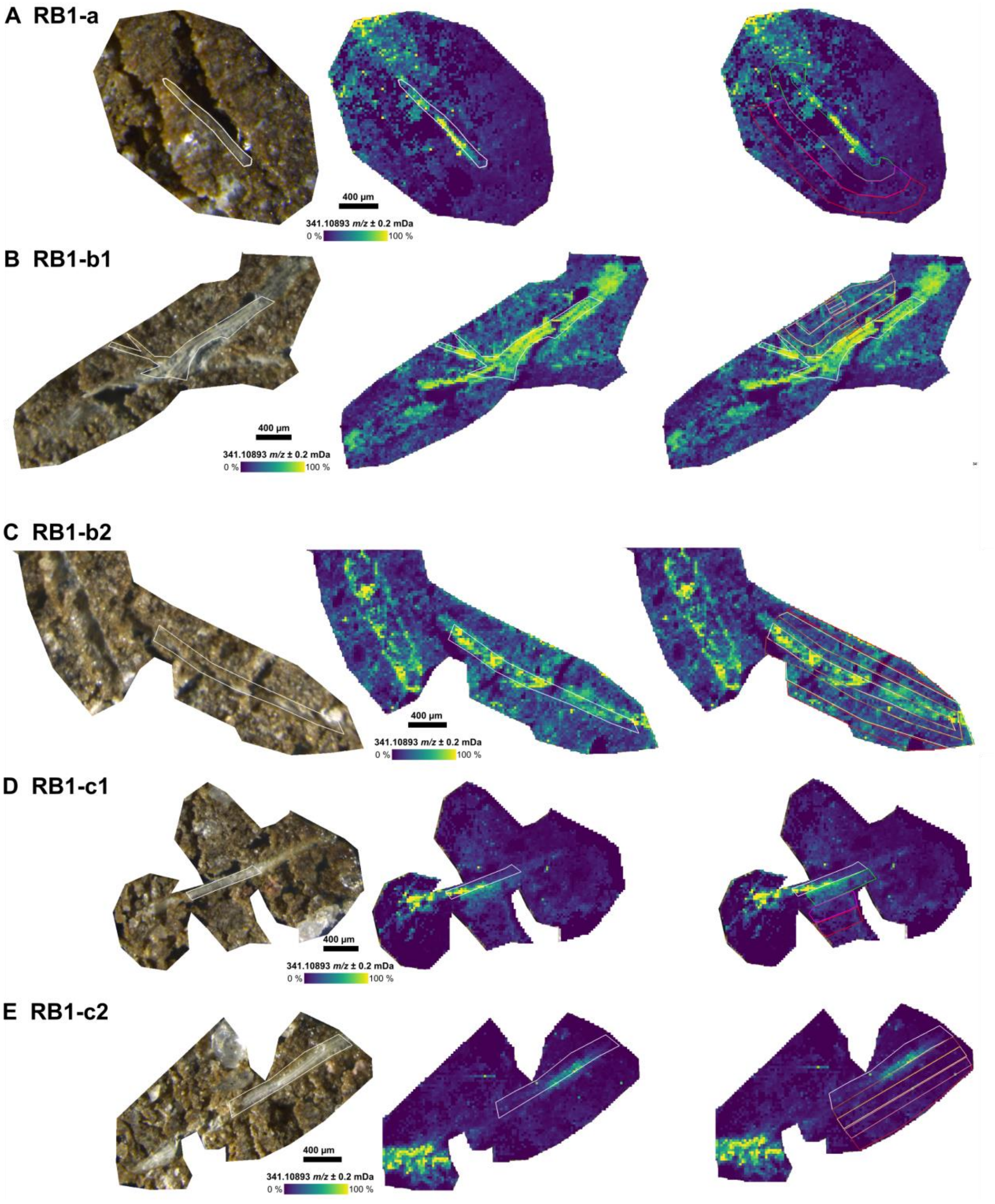

Figure S4: Imaging results for undisturbed soil samples from the laboratory experiment. Optical image of the soil section and ion image for the dihexose signal $[C_{12}H_{22}O_{11}-H]^-$ as detected by LDI-FT-ICR-MSI the root surface is highlighted in white. Right: Ion image for the dihexose signal $[C_{12}H_{22}O_{11}-H]^-$ with the analyzed regions of interest for the rhizosphere gradients (ROI 1 is the root, ROI 2 to ROI 4 or 5 are regions with increasing distance to the root surface).

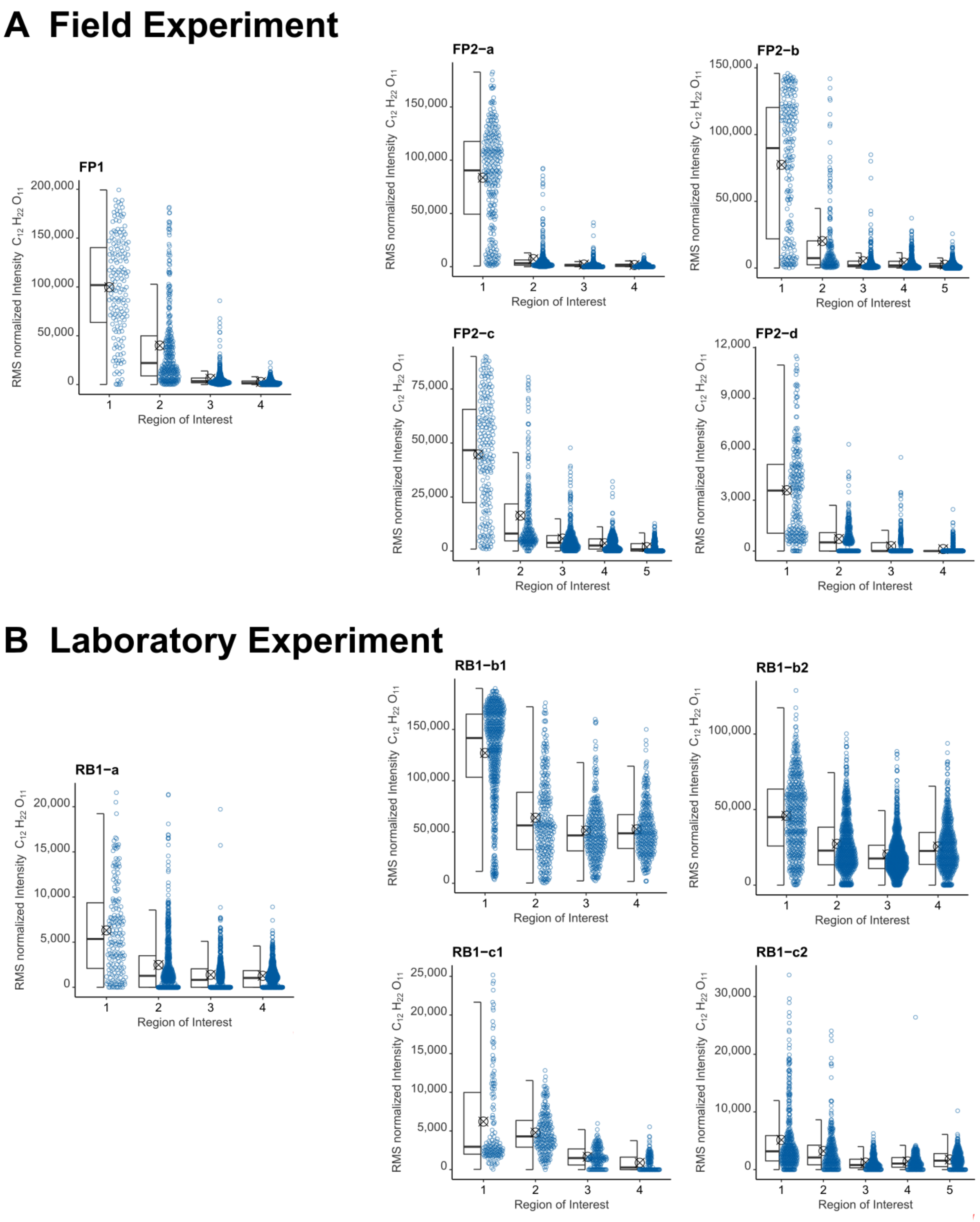

Figure S5: Decrease of the peak intensity of the dihexose signal $[C_{12}H_{22}O_{11}-H]^-$ as detected by LDI-FT-ICR-MSI in regions of interest with increasing maximum distance to the root surface for A) samples from the field experiment and the B) laboratory experiments. ROI 1 shows the intensity detected in the root, ROI 2 to ROI 5 are regions of soil with increasing distance to the root surface. Each spectrum is represented by one blue dot. The areas used to construct the boxplots (symbol indicates arithmetic mean) are shown in Figure S3 and Figure S4 for the field experiment and the laboratory experiments, respectively.

Table S2: Molecular formula annotation for the MSI data of soil section FP2-d (*m/z* 172 ± 10 Da). Summarized are selected molecular formulas localized in the root, the *m/z* of the $[M-H]^-$, the false discovery rate, and possible metabolites as annotated by METASPACE (Palmer et al., 2017) using the ChEBI-Database.

**Molecular Formula**	***m/z* $[M-H]^-$**	**False Discovery Rate**	**Possible Metabolites**
$C_9H_8O_3$	163.0401	0.05	coumaric acid (Azaizeh et al., 1995; Seal et al., 2004; Zhang et al., 2020)
$C_9H_{10}O_3$	165.0557	0.05	phenyllactic acid (Schilling et al., 1985), caffeyl alcohol, tropic acid, phloretic acid, ethylvanillin, apocynin, veratraldehyde, ethoxybenzoic acid
$C_8H_8O_4$	167.0350	0.05	vanillic acid (Azaizeh et al., 1995; Seal et al., 2004; Zhang et al., 2020)
$C_9H_8O_4$	179.0350	0.05	caffeic acid (Seal et al., 2004)
$C_6H_{12}O_6$ *	179.0561	0.1	glucose/ fructose (Walter et al., 2003; Fan et al., 2012), mannose/ inositol (Fan et al., 2012)

* was annotated by using the Human Metabolome Database (v4).

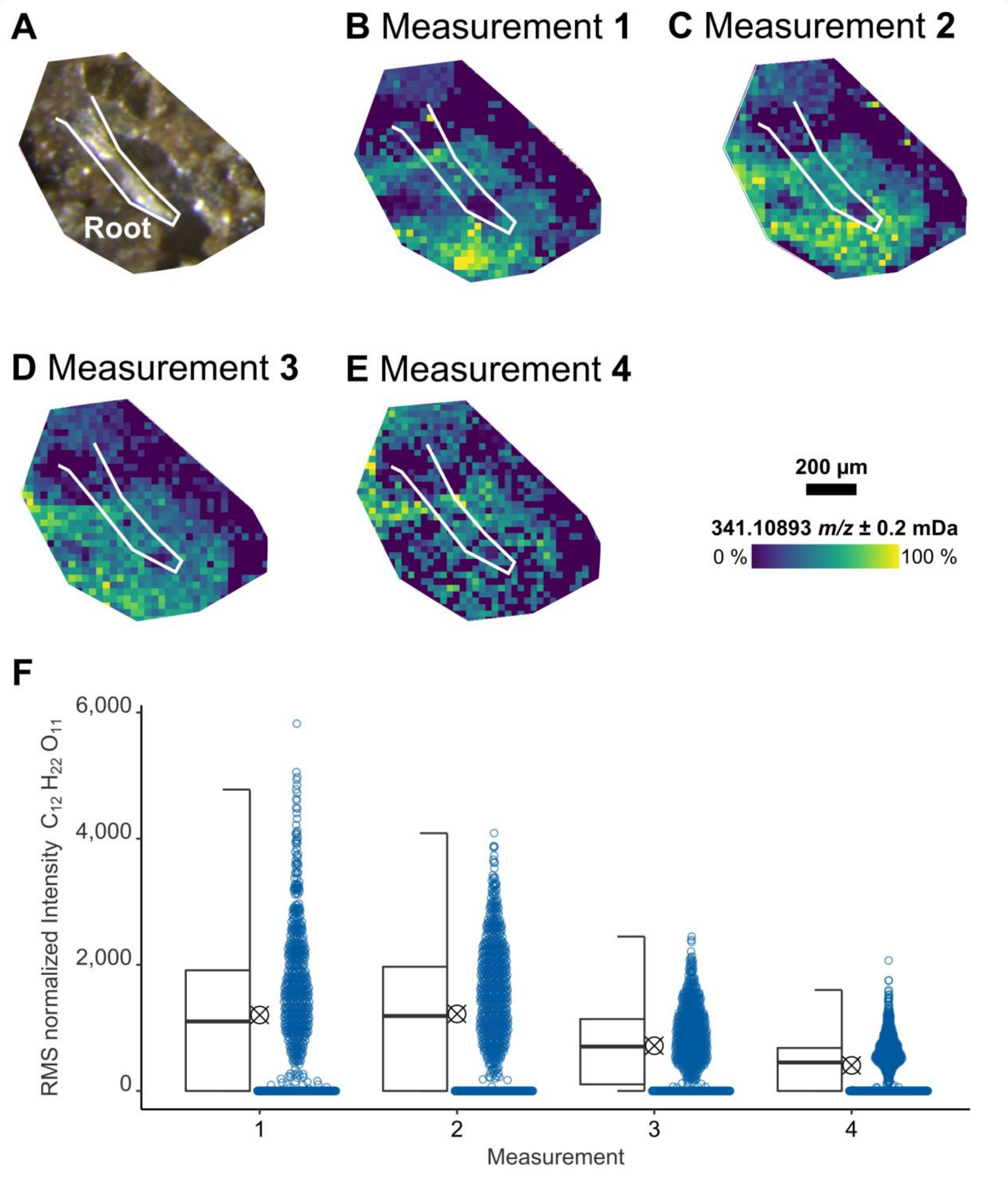

Figure S6: Repeated measurement of the same region on a soil section. A) Optical image of the soil section RB2. B) to E) Ion image for the dihexose signal $[C_{12}H_{22}O_{11}-H]^{-}$ as detected by LDI-FT-ICR-MSI after one, two, three, and four measurements, respectively. F) Decrease of the peak intensity of the dihexose signal $[C_{12}H_{22}O_{11}-H]^{-}$ in the same ROI for multiple measurements. Each spectrum is represented by one blue dot. The complete area was used to construct the boxplots (symbol indicates arithmetic mean).

Table S3: Molecular formula annotation for the MSI data of soil section FP1 (*m/z* 341.5 ± 5 Da). Summarized are all molecular formulas localized in the root, the *m/z* of the $[M-H]^-$, the false discovery rate, and the co-localization value with the dihexose ($C_{12}H_{22}O_{11}$) as annotated by METASPACE (Palmer et al., 2017) using the ChEBI-Database.

**Molecular Formula**	***m/z* $[M-H]^-$**	**False Discovery Rate**	**Co-localization value ($C_{12}H_{22}O_{11}$)**
$C_6H_{10}O_5$ *	161.0455	0.1	0.75
$C_6H_{12}O_6$ *	179.0561	0.1	0.84
$C_{19}H_{16}O_6$	339.0874	0.1	0.76
$C_{19}H_{18}O_6$	341.1031	0.1	0.76
$C_{12}H_{22}O_{11}$	341.1089	0.1	1
$C_{19}H_{20}O_6$	343.1187	0.05	0.76
$C_{18}H_{18}O_7$	345.0980	0.1	0.59

* Possible fragments of saccharides (e.g. $C_{12}H_{22}O_{11}$) see also Figure S7.

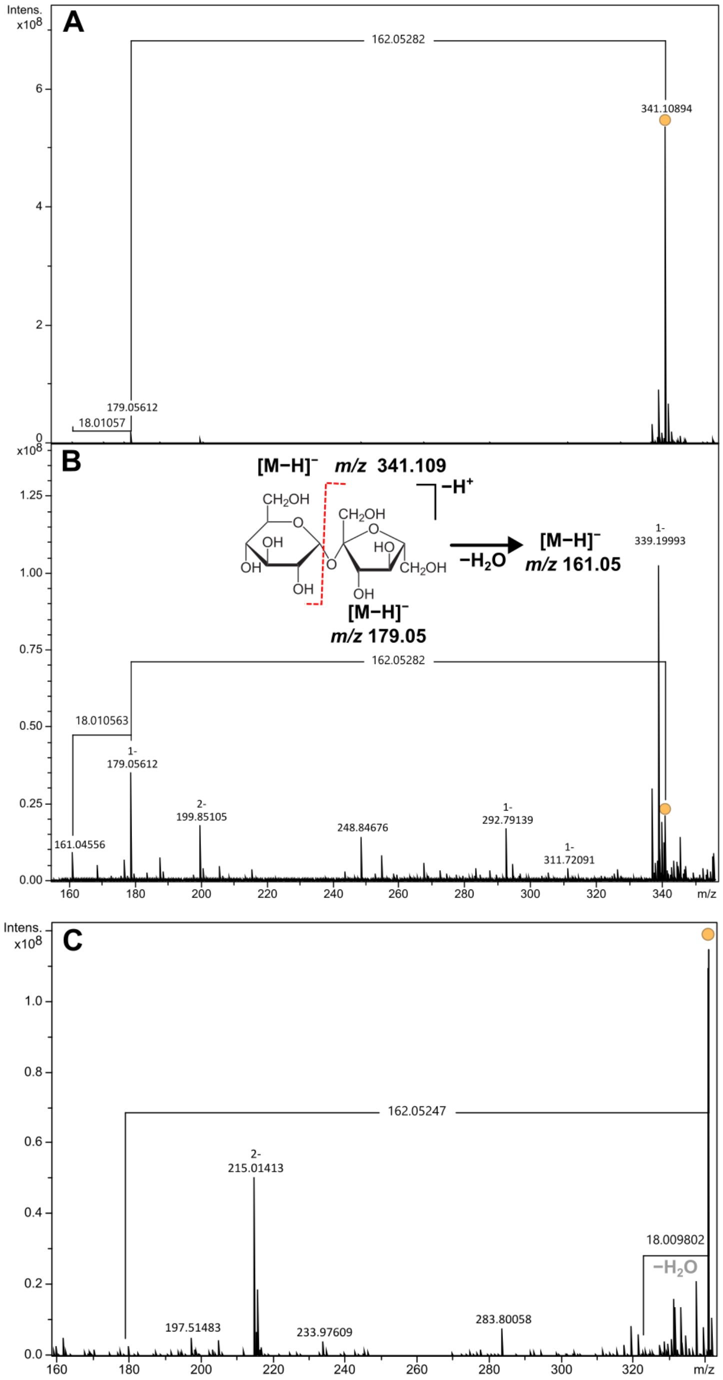

Figure S7: LDI-CID-FT-ICR-MS/MS experiment of the sucrose standard. The sucrose signal $[C_{12}H_{22}O_{11}-H]^-$ is highlighted by an orange dot. A) Isolation spectrum and B) fragmentation spectrum (15 V collision voltage) of a 10 ppm sucrose standard. The cleavage of the glycosidic bond with an additional loss of water from the resulting fragment could be observed with higher intensity in the fragmentation experiment (Calvano et al., 2017). The same fragments with lower intensity were already observed without using a collision voltage indicating the effect of ion-ion interactions in the hexapol or the ICR cell. C) Average spectrum of 47 scans of a 1000 ppm sucrose standard, analyzed without CASI-isolation (full scan, 70% laser power). Only a low intensity of fragment ions was observed.

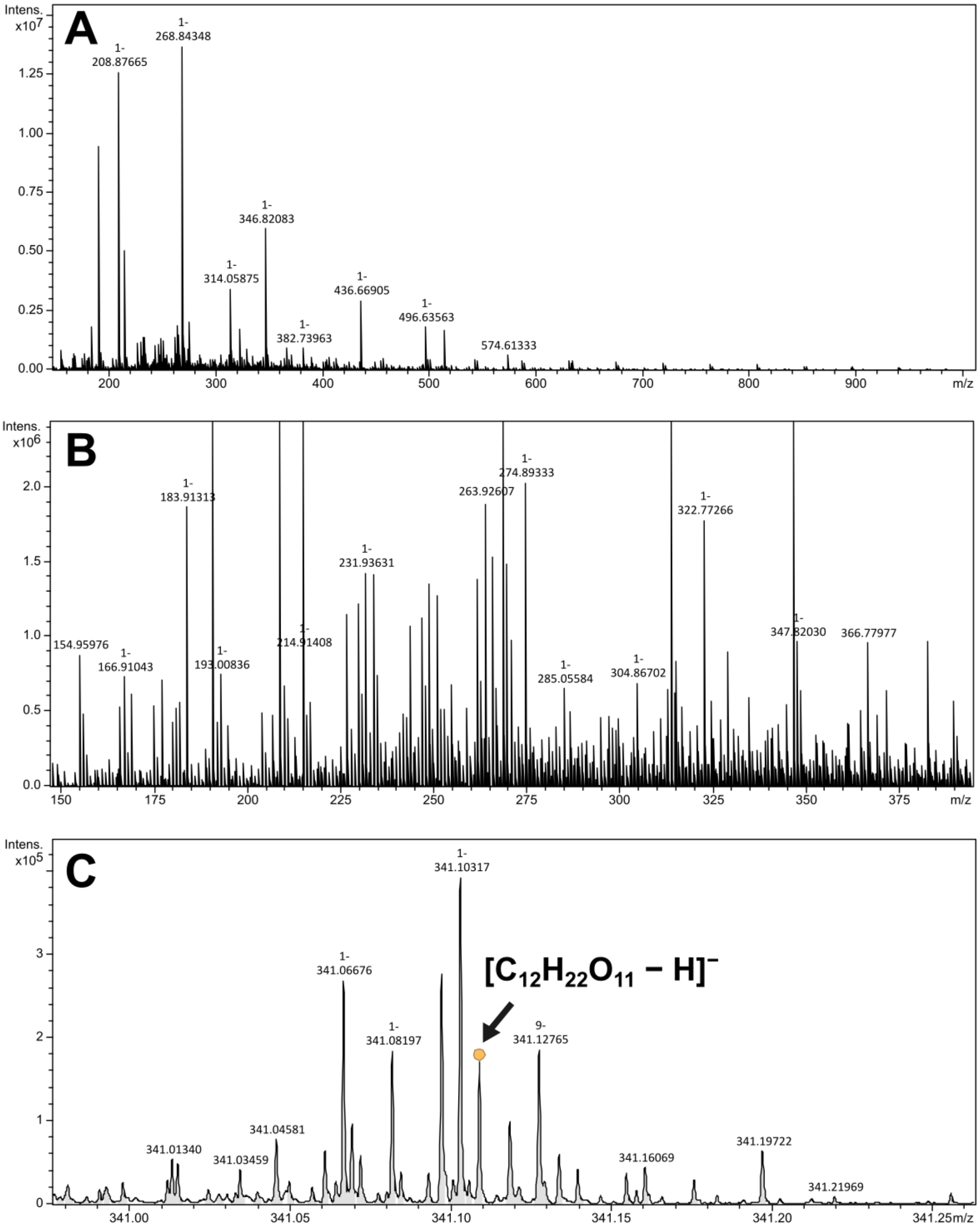

Figure S8: Zoom into an LDI-FT-ICR-MS mass spectrum averaged for the whole analyzed region of a soil section (FP2-d) without applying the CASI mode (full scan spectrum). A) Complete mass window 147 – 1000 Da. B) Zoom into the mass region < 400 *m/z* reveals a high number of signals, C) Zoom into the nominal mass of the dihexose ($[C_{12}H_{22}O_{11}-H]^-$, *m/z* 341.1089, highlighted by an orange dot) shows the chemical complexity detectable on the nominal mass level indicating ionization of soil organic matter.

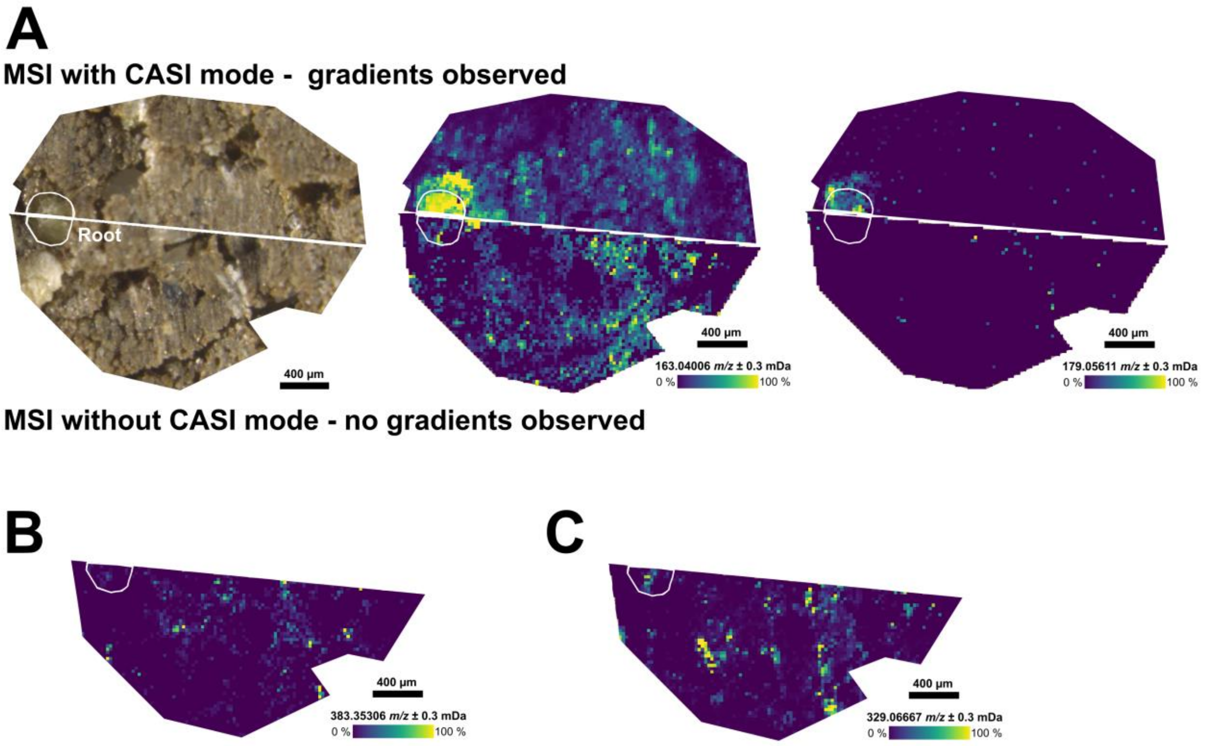

Figure S9: Localization of plant metabolites without applying the CASI mode is not possible. A) Optical image and two ion images ($[C_9H_8O_3{-}H]^-$ *m/z* 163.04006, e.g coumaric acid and $[C_6H_{12}O_6{-}H]^-$ *m/z* 179.05611, e.g. glucose) of two measurement regions on the same soil section (FP2-d) **-** the upper half was analyzed with CASI, the lower half without CASI. B) and C) Two ion images of the only masses annotated by METASPACE (Palmer et al., 2017) using the ChEBI-Database without applying the CASI mode. B) $[C_{24}H_{48}O_3{-}H]^-$ (*m/z* 383.3531), C) $[C_{17}H_{14}O_7{-}H]^-$ (*m/z* 329.0667). No spatial correlation to the position of the root was observed.

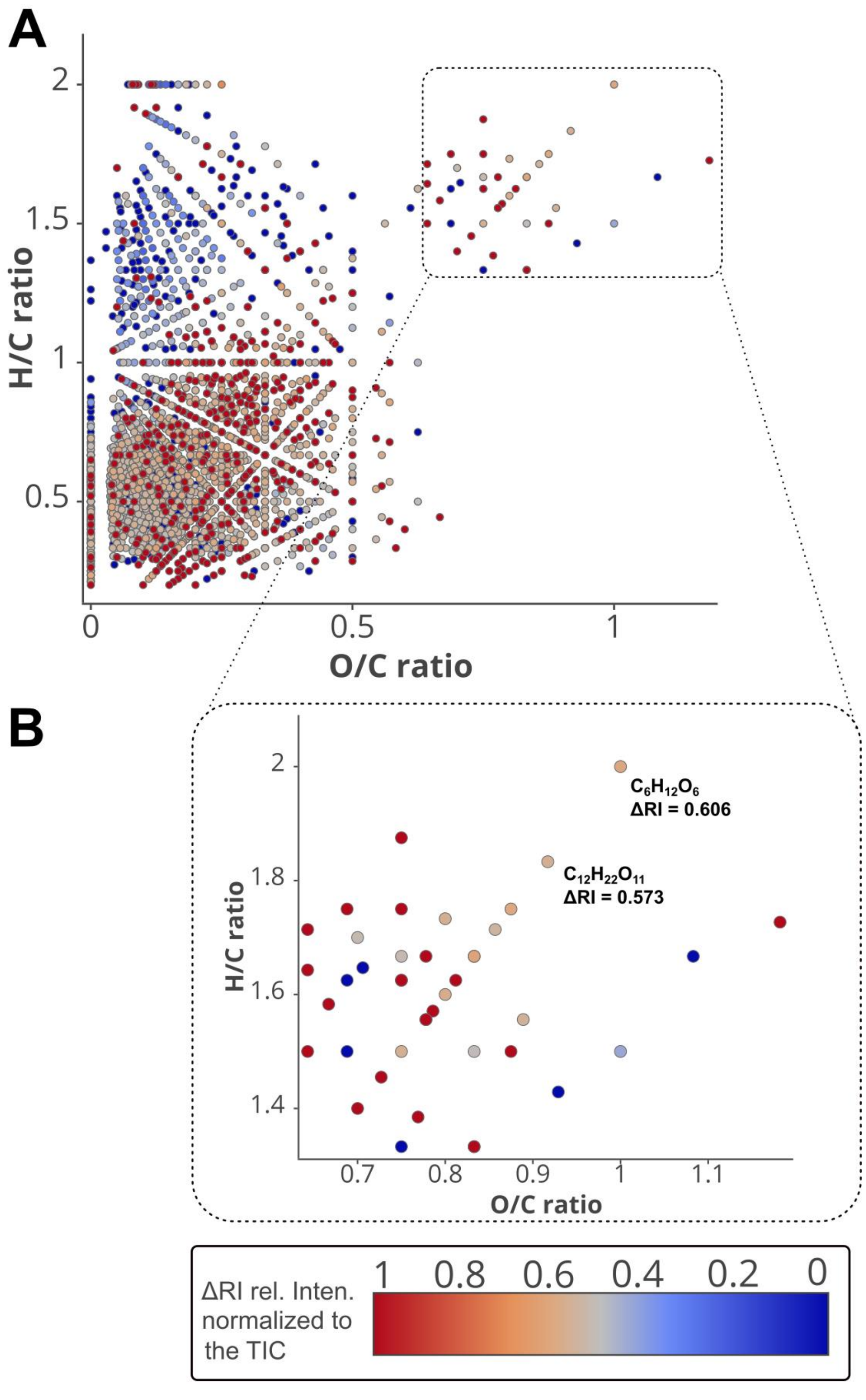

Figure S10: Comparison of the intensity ratio for disturbed bulk- and rhizosphere soil samples using LDI-FT-ICR-MS. A) Overlay of van Krevelen diagrams. Comparisons are shown for the mean of three replicates for each bulk soil (BS, blue) and rhizosphere soil (RS, red). Molecular formulas unique for either BS or RS are shown in dark blue or dark red, respectively. Molecular formulas, where the TIC normalized relative intensity is more than 50% higher intensity in one compartment, are colored either blue or red. Molecular formulas with no significant difference in the intensity are shown in grey. B) Zoom into the region of the van Krevelen diagram with O/C > 0.7 and H/C > 1.3 where putative sugars are detected.

Table S4: Aggregated molecular parameters for disturbed bulk - and rhizosphere soil samples after isolation of the soil from the root and direct measurement of soil without embedding via LDI-FT-ICR-MS. Compared is the mean for molecular formulas present in three replicates from the same laboratory experiment.

**Sample**	**Bulk soil**	**Rhizosphere soil**
Number CHO formulas	969	961
Number CHNO formulas	590	715
Number CHNOS formulas	0	1
Number CHOS formulas	7	13
Number CHN formulas	76	73
Number CHS formulas	0	1
Number other formulas	69	57
CHO %	56.63	52.77
CHNO %	34.48	39.26
CHNOS %	0	0.05
CHOS %	0.41	0.71
CHN %	4.44	4.01
CHS %	0	0.05
other %	4.03	3.13
formulas assigned	1711	1821
mean *m/z*	280.90	282.09
mean H/C-ratio	0.76	0.73
mean O/C-ratio	0.19	0.21
mean N/C-ratio	0.03	0.04
mean S/C-ratio	0.00	0.00
mean DBE	12.44	12.65
mean DBE-O	9.36	9.36
wa *m/z*	215.97	215.87
wa H/C-ratio	0.67	0.56
wa O/C-ratio	0.14	0.16
wa N/C-ratio	0.02	0.02
wa S/C-ratio	0.00	0.00
wa DBE	8.82	9.73
wa DBE-O	6.51	7.16

Table S5: Exact masses and natural abundance for the isotopologues of the ion formula of a dihexose [$C_{12}H_{22}O_{11}$ − H]$^-$ Calculated with Compass IsotopePattern (Bruker Daltonics).

**Exact *m/z* [M−H]$^-$**	**Natural abundance [%]**	**Isotopologue**
341.108935	100	Monoisotopic [$^{12}C_{12}{}^{1}H_{22}{}^{16}O_{11}$− ^{1}H]$^-$
342.11229	12.979	$^{13}C_1$
342.113152	0.419	$^{17}O_1$
342.115212	0.242	$^{2}H_1$
343.11318	2.26	$^{18}O_1$
343.115645	0.772	$^{13}C_2$
343.116507	0.054	$^{13}C_1{}^{17}O_1$
343.118567	0.031	$^{13}C_1{}^{2}H_1$
344.116535	0.293	$^{13}C_1{}^{18}O_1$
344.119	0.028	$^{13}C_3$
345.117425	0.023	$^{18}O_2$
345.11989	0.017	$^{13}C_2{}^{18}O_1$

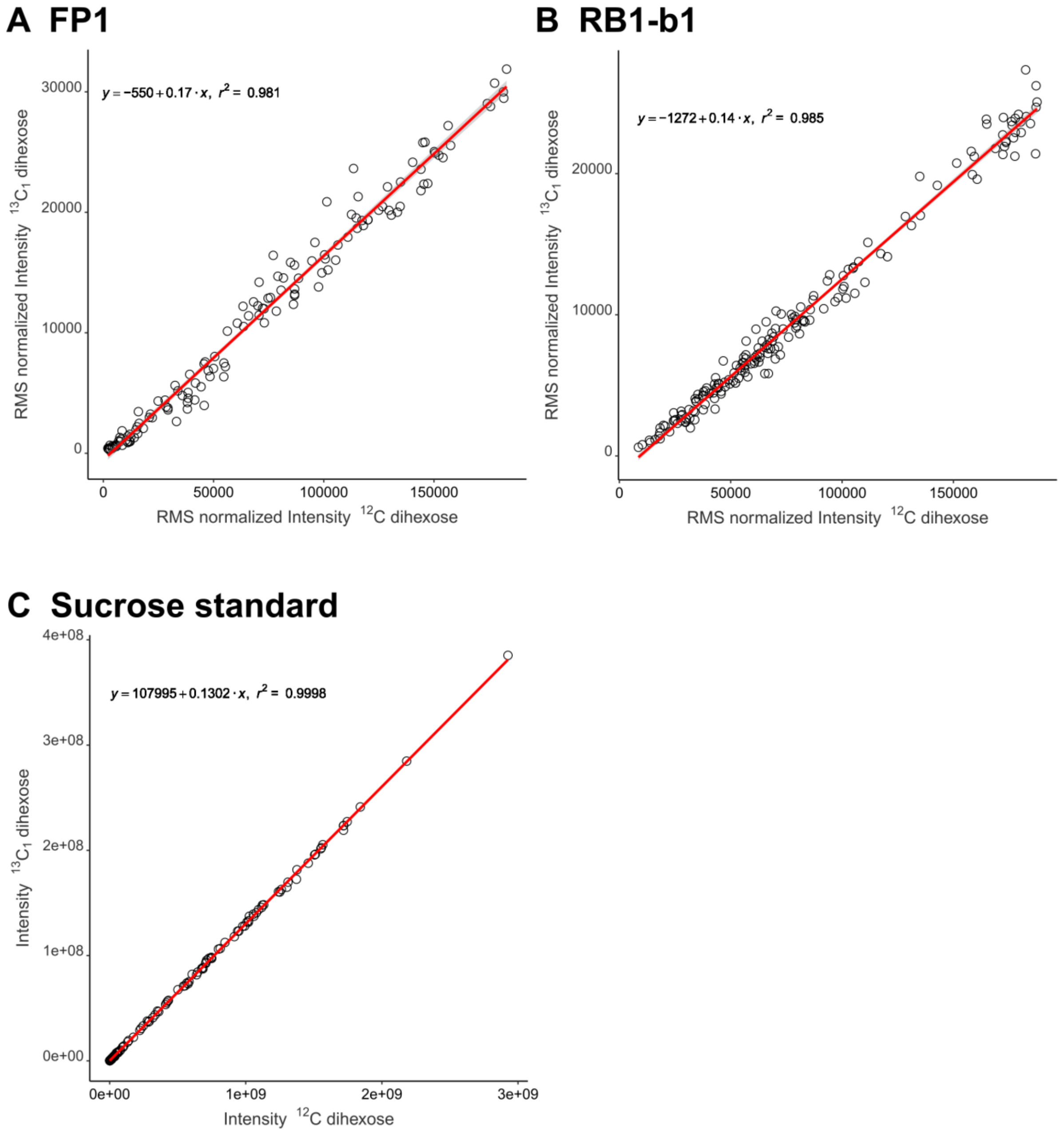

Figure S11: Correlation plots for the $^{13}C_1$ and ^{12}C intensity of the dihexose signal $[C_{12}H_{22}O_{11}-H]^-$ for the LDI-FT-ICR-MSI. $^{13}C_1$ and ^{12}C intensities are shown for all laser spots with detectable $^{13}C_1$-Signal. A) ROI for an undisturbed soil sample from the field experiment (FP1). B) ROI for an undisturbed soil sample from a laboratory experiment (RB1-b1). C) Sucrose standard: three concentrations of a sucrose standard were spotted on a steel target and analyzed by LDI-FT-ICR-MS with different laser power (measured spots for each setting: n = 39). The slope of the regression depends on the $^{13}C_1/^{12}C$ isotope ratio (natural abundance: 0.1298 for $[C_{12}H_{22}O_{11}-H]^-$)

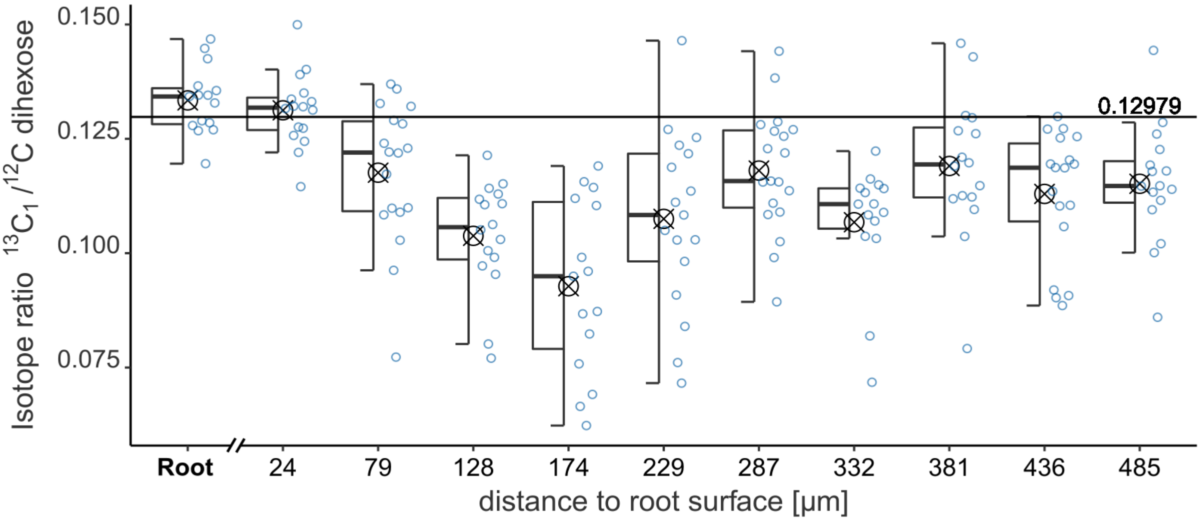

Figure S12: Isotope ratio for an undisturbed soil sample from a laboratory experiment (RB1-b1) as detected by LDI-FT-ICR-MSI. Isotope ratio for regions versus maximum distance to the root surface considering the $^{13}C_1/^{12}C$ ratio of the dihexose $[C_{12}H_{22}O_{11}-H]^-$ for all laser spots with detectable $^{13}C_1$-Signal (natural abundance: 0.1298). The isotope ratio for each laser spot is shown as a blue circle. The areas used to construct the boxplots (symbol indicates arithmetic mean) are highlighted in the ion image in Figure 1 B. No ^{13}C pulse labeling was conducted during the laboratory experiments.

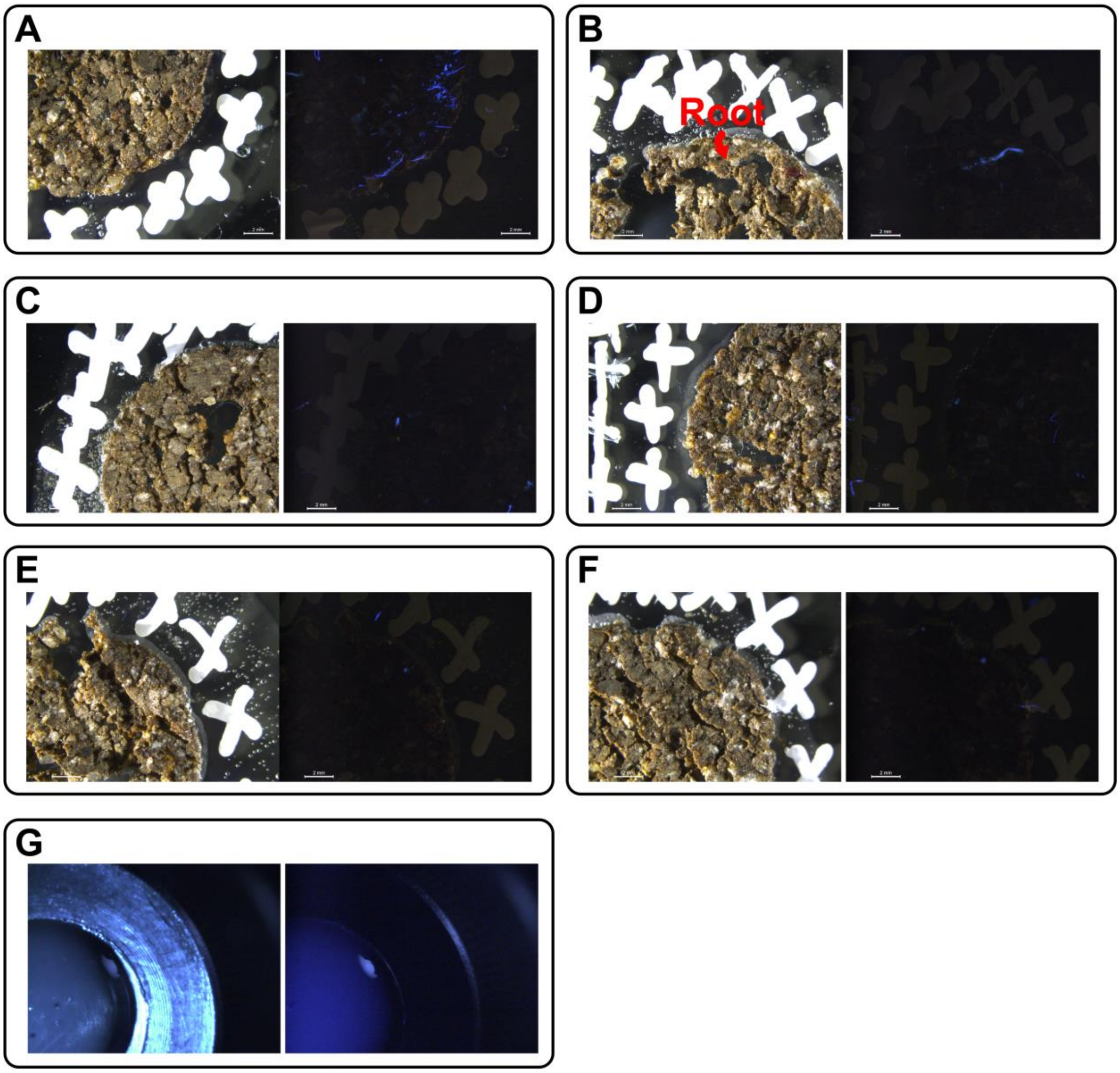

Figure S13: Optical (left) and UV-fluorescence images (right) of soil sections and the embedding medium. Panels A) to F) show images of soil sections. In A) the fluorescence of the embedding medium can be seen in bigger pores and the edge of the soil section which represents a non-ideal case for the MSI analysis. The fluorescence of the embedding medium could not be detected on other soil sections (B to F), but the autofluorescence of the root is clearly visible in B). G) Optical image of a drop of the embedding medium (left) and the fluorescence during UV excitation (right).

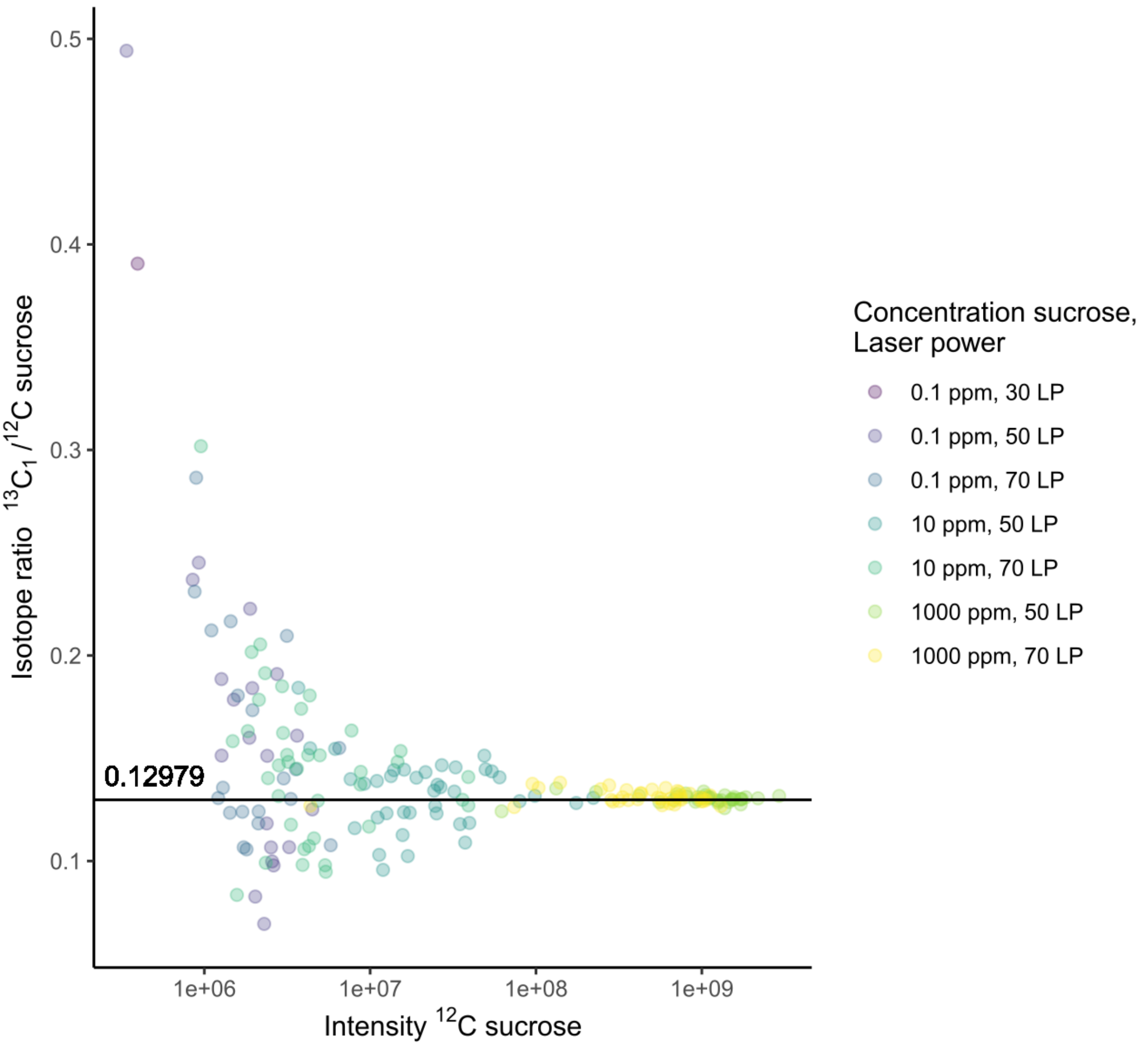

Figure S14: Dependency of the $^{13}C_1/^{12}C$ ratio of sucrose $[C_{12}H_{22}O_{11}{-}H]^-$ on the ^{12}C-intensity. Three concentrations of a sucrose standard were spotted on a steel target and analyzed by LDI-FT-ICR-MS with different laser power (LP, measured spots for each setting: n = 39). $^{13}C_1/^{12}C$ ratio shown for all laser spots with detectable $^{13}C_1$-Signal. With increasing intensity of the ^{12}C-signal, the detected $^{13}C_1/^{12}C$ isotope ratio converges towards the natural abundance (0.1298 for $[C_{12}H_{22}O_{11}{-}H]^-$).

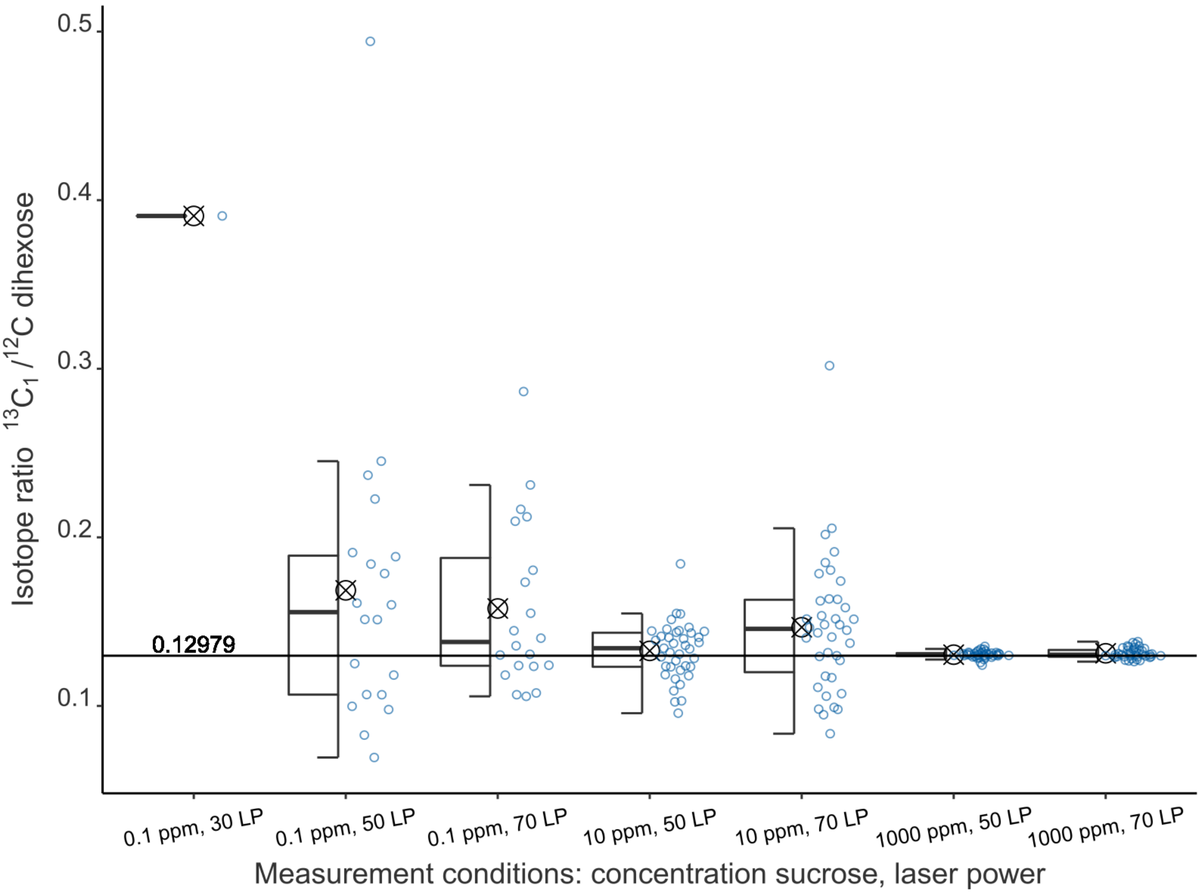

Figure S15: $^{13}C_1/^{12}C$ ratio of sucrose $[C_{12}H_{22}O_{11}-H]^-$ for different measurement conditions. Three concentrations (0.1, 10, 1000 ppm) of a sucrose standard were spotted on a steel target and analyzed by LDI-FT-ICR-MS with different laser power (LP) (measured spots for each setting: n = 39). $^{13}C_1/^{12}C$ ratio of $[C_{12}H_{22}O_{11}-H]^-$ was calculated for all laser spots with detectable $^{13}C_1$-Signal (natural abundance: 0.1298). The isotope ratio for each laser spot is shown as a blue circle and aggregated as boxplots (symbol indicates arithmetic mean).

Supplementary References

Azaizeh, H. A., Marschner, H., Römheld, V., and Wittenmayer, L. (1995). Effects of a vesicular-arbuscular mycorrhizal fungus and other soil microorganisms on growth, mineral nutrient acquisition and root exudation of soil-grown maize plants. *Mycorrhiza* 5, 321–327. doi: 10.1007/BF00207404

Calvano, C. D., Cataldi, T. R. I., Kögel, J. F., Monopoli, A., Palmisano, F., and Sundermeyer, J. (2017). Structural Characterization of Neutral Saccharides by Negative Ion MALDI Mass Spectrometry Using a Superbasic Proton Sponge as Deprotonating Matrix. *J. Am. Soc. Mass Spectrom.* 28, 1666–1675. doi: 10.1007/s13361-017-1679-y

Fan, B., Carvalhais, L. C., Becker, A., Fedoseyenko, D., Wirén, N. von, and Borriss, R. (2012). Transcriptomic profiling of *Bacillus amyloliquefaciens* FZB42 in response to maize root exudates. *BMC Microbiol.* 12, 116. doi: 10.1186/1471-2180-12-116

Herzsprung, P., Hertkorn, N., Tümpling, W. von, Harir, M., Friese, K., and Schmitt-Kopplin, P. (2014). Understanding molecular formula assignment of Fourier transform ion cyclotron resonance mass spectrometry data of natural organic matter from a chemical point of view. *Anal. Bioanal. Chem.* 406, 7977–7987. doi: 10.1007/s00216-014-8249-y

Kind, T., and Fiehn, O. (2007). Seven Golden Rules for heuristic filtering of molecular formulas obtained by accurate mass spectrometry. *BMC Bioinform.* 8, 105. doi: 10.1186/1471-2105-8-105

Koch, B. P., Dittmar, T., Witt, M., and Kattner, G. (2007). Fundamentals of molecular formula assignment to ultrahigh resolution mass data of natural organic matter. *Anal. Chem.* 79, 1758–1763. doi: 10.1021/ac061949s

Koch, B. P., Kattner, G., Witt, M., and Passow, U. (2014). Molecular insights into the microbial formation of marine dissolved organic matter: recalcitrant or labile? *Biogeosciences* 11, 4173–4190. doi: 10.5194/bg-11-4173-2014

Lechtenfeld, O. J., Kattner, G., Flerus, R., McCallister, S. L., Schmitt-Kopplin, P., and Koch, B. P. (2014). Molecular transformation and degradation of refractory dissolved organic matter in the Atlantic and Southern Ocean. *Geochim. Cosmochim. Acta* 126, 321–337. doi: 10.1016/j.gca.2013.11.009

Palmer, A., Phapale, P., Chernyavsky, I., Lavigne, R., Fay, D., Tarasov, A., et al. (2017). FDR-controlled metabolite annotation for high-resolution imaging mass spectrometry. *Nat. Methods* 14, 57–60. doi: 10.1038/nmeth.4072

Schilling, D. G., Liebl, R. A., and Worsham, A. D. (1985). “Rye (*Secale cereale* L.) and Wheat (*Triticum aestivum* L.) Mulch: The Suppression of Certain Broadleaved Weeds and the Isolation and Identification of Phytotoxins,” in *The Chemistry of Allelopathy: Biochemical Interactions Among Plants*, ed. A. C. Thompson (Washington, D.C., USA: American Chemical Society), 243–271.

Seal, A. N., Pratley, J. E., Haig, T., and An, M. (2004). Identification and quantitation of compounds in a series of allelopathic and non-allelopathic rice root exudates. *J. Chem. Ecol.* 30, 1647–1662. doi: 10.1023/b:joec.0000042074.96036.14

Walter, A., Feil, R., and Schurr, U. (2003). Expansion dynamics, metabolite composition and substance transfer of the primary root growth zone of *Zea mays* L. grown in different external nutrient availabilities. *Plant Cell Environ.* 26, 1451–1466. doi: 10.1046/j.0016-8025.2003.01068.x

Zhang, H., Yang, Y., Mei, X., Li, Y., Wu, J., Li, Y., et al. (2020). Phenolic Acids Released in Maize Rhizosphere During Maize-Soybean Intercropping Inhibit

Phytophthora Blight of Soybean. *Front. Plant Sci.* 11, 886. doi: 10.3389/fpls.2020.00886
